# Skewed T cell responses to Epstein-Barr virus in long-term asymptomatic kidney transplant recipients

**DOI:** 10.1371/journal.pone.0224211

**Published:** 2019-10-22

**Authors:** Cecilia Nakid-Cordero, Nadia Arzouk, Nicolas Gauthier, Nadine Tarantino, Martin Larsen, Sylvain Choquet, Sonia Burrel, Brigitte Autran, Vincent Vieillard, Amélie Guihot

**Affiliations:** 1 Sorbonne Université (Univ. Paris 06), INSERM U1135, Centre d'Immunologie et des Maladies Infectieuses (CIMI-Paris), Hôpital Pitié-Salpêtrière, Paris, France; 2 Service de Néphrologie, Urologie et Transplantation Rénale, Hôpital Pitié Salpêtrière, Paris, France; 3 CNRS ERL8255, Centre d'Immunologie et des Maladies Infectieuses (CIMI-Paris), Paris, France; 4 Service d’Hématologie, Hôpital Pitié Salpêtrière, Paris, France; 5 Service de Virologie, Hôpital Pitié Salpêtrière, Paris, France; 6 Département d’Immunologie, Assistance Publique-Hôpitaux de Paris (AP-HP), Groupe Hospitalier Pitié-Salpêtrière, Paris, France; Mercer University School of Medicine, UNITED STATES

## Abstract

Kidney transplant recipients (KTRs) abnormally replicate the Epstein Barr Virus (EBV). To better understand how long-term immunosuppression impacts the immune control of this EBV re-emergence, we systematically compared 10 clinically stable KTRs to 30 healthy controls (HCs). The EBV-specific T cell responses were determined in both groups by multiparameter flow cytometry with intra cellular cytokine staining (KTRs n = 10; HCs n = 15) and ELISpot-IFNγ assays (KTRs n = 7; HCs n = 7). The T/B/NK cell counts (KTRs n = 10; HCs n = 30) and the NK/T cell differentiation and activation phenotypes (KTRs n = 10; HCs n = 15/30) were also measured. We show that in KTRs, the Th1 effector CD4^+^ T cell responses against latent EBV proteins are weak (2/7 responders). Conversely, the frequencies total EBV-specific CD8^+^T cells are conserved in KTRs (n = 10) and span a wider range of EBNA-3A peptides (5/7responders) than in HCs (5/7responders). Those modifications of the EBV-specific T cell response were associated with a profound CD4^+^ T cell lymphopenia in KTRs compared to HCs, involving the naïve CD4^+^ T cell subset, and a persistent activation of highly-differentiated senescent CD8^+^ T cells. The proportion of total NK / CD8^+^ T cells expressing PD-1 was also increased in KTRs. Noteworthy, PD-1 expression on CD8^+^ T cells normalized with time after transplantation. In conclusion, we show modifications of the EBV-specific cellular immunity in long term transplant recipients. This may be the result of both persistent EBV antigenic stimulation and profound immunosuppression induced by anti-rejection treatments. These findings provide new insights into the immunopathology of EBV infection after renal transplantation.

## Introduction

Epstein Barr Virus (EBV) infection is usually asymptomatic in the immunocompetent[1]. After kidney engraftment, therapeutic immunosuppression renders patients vulnerable to infections, often leading to EBV abnormal replication in peripheral blood, a virological status that predispose to EBV-induced post-transplant lymphoproliferative disorders (PTLD).[2–4] EBV-related PTLDs mainly arises early after kidney transplantation but can also occur in the long term.[4] EBV replication is known to be controlled by both T cells [5], and NK cells [6,7]. Thus, analyzing how long-term immunosuppression in kidney transplant recipients (KTRs) alters EBV-specific immune response is paramount for preventing long-term EBV-associated complications.

To date, the mechanism leading to the loss of immune control over viral replication after transplantation remains poorly understood. The first mechanisms that could be evoked is immunosuppression with CD4^+^ T cell lymphopenia[8,9]. This loss of immune cells could be linked with activation induced cell death: increased proportions of activated CD38^+^ and pre-apoptotic CD95^+^ T cells have been described in KTRs [10]. Finally, exhausted PD-1^+^ CD8^+^ T cells can be generated in a vicious cycle with viral replication that constitute chronic antigenic stimulation [11,12]. Thus, deciphering the immunopathogenesis of the chronic viral replication in KTRs is paramount if we are to early detect kidney transplant recipients at risk for EBV-related complications.

Such precancerous/cancerous complications are more frequent in multi-organ and thoracic transplant recipients, due to the intensity of maintenance immunosuppression, and in EBV-seronegative recipients, who are mostly infants.[4] Thus, the EBV-specific T cell responses have been mainly studied in those high risk transplant populations. [13–16] In such patients, the effector T CD8^+^ cell responses against EBV seem to be conserved in terms of IFNγ production, when compared to pre-transplant recipients or healthy controls[14,16]. The ability to develop a protective EBV-specific CD8^+^ T cell response has been observed in one EBV seronegative patient who underwent cardiac transplantation from an EBV seropositive donor [17]. However, functional alterations have been reported in children presenting high EBV loads after transplantation, including increased frequencies of monofunctional IFNγ^+^ lytic-EBV specific CD8 T cells[15] and increased expression of PD-1 and CD38 activation markers on EBV-specific T cells, suggesting an exhaustion of those cells.[13] In contrast, the EBV-specific T cell immunity after long-term kidney transplantation have been insufficiently described, especially in the adult population.

Following lung and liver transplantation, immunosuppressive drugs [18] and chronic viral infections [19,20] have been associated with altered NK cell differentiation and function that might expose to non-Hodgkin lymphoma[21,22]. Whether these alterations result from NK cell exhaustion and enhanced PD-1 expression on NK cells, as reported in pediatric PTLD patients, [23] have not yet been studied in adult long-term stable KTRs.

Given that kidney transplantation represents almost half of total solid organ transplantation (SOT) worldwide, according to the Global Observatory on Donation and Transplantation (GODT) data, produced by the WHO-ONT collaboration (http://www.transplant-observatory.org), the number of cases of EBV-related complications is probably as elevated for adult KTRs as for other transplanted populations at higher risk. Yet, the EBV-specific cellular immunity has been poorly described in KTRs. Thus, to better understand the EBV long-term immune control after adult kidney transplantation, we conducted a systematic study of peripheral immune cells in long-term clinically stable KTRs and compared them to healthy controls (HCs). Our results show that in KTRs, the Th1 effector CD4^+^T cell responses against latent EBV proteins are weak (2/7 responders). In contrast, the frequencies of EBV-specific CD8^+^T cells are conserved in KTRs (n = 10) and patients responding (n = 5/7) to EBNA-3A, span a wider range of the protein peptide sequence than responding HCs (n = 2/7).

## Materials and methods

### Study groups

This retrospective study, performed in 2016, included 10 long-term clinically stable kidney transplant recipients (KTRs) from the renal transplantation department of the Pitié-Salpêtrière Hospital (Paris, France). Inclusion criteria were: age >18 years old at transplantation, post-transplant follow-up ≥2 years after blood sample, no history of acute graft rejection, malignancy or recurrent infections after transplantation. We selected patients with different post-transplant delays in order to study immune alterations through time. Blood samples for patients were obtained in the context of follow-up consultation and clinical routine blood withdrawal. The healthy control (HC) group was composed of 30 anonymous adult healthy donors, from the French blood bank (Etablissement Français du Sang, Paris, France).

The study was approved by institutional research ethics board, Comité de Protection des Personnes Ile de France VII, under the protocol no° PP13-022, and performed in accordance with the human-experimentation guidelines of the Pitié-Salpêtrière Hospital and the declaration of Helsinki, as well as respecting the convention signed between the INSERM (Institut National de la Santé Et de la Recherche Medicale, Ile-de-France, France) and the Etablissement Français du Sang (EFS, Ile-de-France, France) for the cession of blood components to research (Reference: C CPSL UNT—N° 15/EFS/012*)*. All patients and healthy controls provided written informed consent.

### Blood sample cryopreservation and viral DNA loads

Blood samples from patients and controls were collected in 5 mL tubes containing Ethylenediaminetetraacetic acid (EDTA) or Lithium heparin. EDTA tubes were obtained for each patient (n = 10) and one for each healthy control (n = 30) and at least one Lithium heparin tube was obtained for 10 patients and 15 healthy controls. Blood collected in Lithium heparin tubes was diluted 1:2 in RPMI 1640 medium (Thermo Fisher Scientific, Villebon-sur-Yvette, Courtaboeuf, France) before 30 minutes centrifugation at 2200 revolutions per minute (rpm) through Lymphocyte separation density gradient media (Eurobio, les Ulis, Courtaboeuf, France). The ring containing the peripheral blood mononuclear cells (PBMC) was recovered, washed twice with 50mL of RPMI 1640 medium for 8 minutes at 1700rpm, then suspended in 10% Dimethyl Sulfoxide (DMSO, Sigma-Aldrich, Missouri, USA) heat-inactivated Fetal Bovine Serum (FBS, Biowest, Nuaillé, France). Samples were cryopreserved in liquid nitrogen cell bank until use. Following experiments were performed according to PBMC availability.

EBV and CMV viral DNA loads were measured in whole blood using commercial kits artus^®^EBV Virus QS-RGQ and artus^®^CMV Virus QS-RGQ (QIAgen, Courtaboeuf, France) according to manufacturer’s instructions, with a detection limit set at ≥1.4 log copies/mL. Human herpes virus 8 (HHV-8) viral DNA loads were measured in fresh peripheral blood mononuclear cells (PBMCs) as described elsewhere,[24] with a detection limit of ≥10 copies/10^6^ cells.

### Absolute lymphocyte counts, T Cell and NK Cell detailed phenotypes

Total and T/B/NK lymphocyte absolute counts were determined on fresh blood, collected in EDTA tubes, with an automated AQUIOS CL flow cytometry system (Beckman Coulter, Villepinte, France) using commercial kits AQUIOS Tetra-1 Panel (ref: B23533) and Tetra-2+ Panel (B23534) according to manufacturer’s instructions (Beckman Coulter).

Detailed T cell phenotype was determined on fresh blood. Briefly, 100μL of blood were stained with two antibody panels shown in Table 1. Anti-CCR7 antibody was incubated at room temperature in the dark for 10 minutes. Then, the rest of antibodies were added and incubated 10 minutes in the same conditions. A TQ-Prep Workstation (Beckman Coulter) was used for incubation, cell lysis and fixation of samples (IMMUNOPREP^™^ Reagent System, Beckman Coulter). A Navios flow cytometer was used for acquisition (Beckman Coulter).

**Table 1:** Primary antibodies used for T cell detailed phenotype.

Antigen	Conjugate	Host	Isotype	Supplier	Catalogue number	Clone	Volume /100μL of blood
**CD57**	FITC	Mouse	IgM	Beckman Coulter, Villepinte, France	B49188	NC1	10μL
**CD45 RA**	ECD	Mouse	IgG1	Beckman Coulter, Villepinte, France	B49193	2H4LDH1 1LDB9 (2H4)	10μL
**CCR7**	PE	Mouse	IgG2A	Agilent Technologies, Les Ulis, France	FAB197P	150503	10μL
**CD95**	FITC	Mouse	IgG1, κ	BD Biosciences, Le Pont de Claix, France	555673	DX2	10μL
**CD25**	PE	Mouse	IgG2A	Beckman Coulter, Villepinte, France	A07774	B1.49.9	10μL
**HLA-DR**	ECD	Mouse	IgG1	Beckman Coulter, Villepinte, France	B92438	Immu-357	10μL
**CD38**	PC7	Mouse	IgG1	Beckman Coulter, Villepinte, France	B49198	LS198-4-3	10μL
**CD3**	APC	Mouse	IgG1	BD Biosciences, Le Pont de Claix, France	345767	SK7 (also known as Leu-4)	5μL
**CD45**	APC-A750	Mouse	IgG1	Beckman Coulter, Villepinte, France	A79392	J33	10μL
**CD4**	Pacific Blue	Mouse	IgG1	Beckman Coulter, Villepinte, France	B49197	13B8.2	10μL
**CD8**	APC-A700	Mouse	IgG1	Beckman Coulter, Villepinte, France	B49181	B9.11	10μL

For the phenotypic study of NK cells, PBMCs were thawed in RPMI+ medium (100 UI/mL penicillin / 100 μg/mL streptomycin / 0.25 μg/mL Amphotericin B, 1 mM sodium pyruvate, 0.1 mM MEM NEAA, 2mM L-glutamine; Thermo Fisher Scientific) supplemented with 20% FBS (Biowest), then washed twice with RPMI+ medium 10% FBS for 5 minutes at 1500rpm. After last wash, 1x10^6^ PBMCs were stained for 30 minutes at 4°C with antibodies described in Table 2. Stained cells were washed with Dulbecco's phosphate-buffered saline (PBS 1x; Thermo Fisher Scientific) and fixed with 300 μL of BD CellFIX 1x (BD Biosciences). At least 5,000 events in the CD3^-^CD56^+^ lymphocytes gate were acquired with a Gallios flow cytometer (Beckman Coulter) and data were analyzed with FlowJo software version 10.5 (Tree star; Ashland, USA). The gating strategy is shown in S1 Fig.

**Table 2:** Primary antibodies used for NK cell detailed phenotype.

Antigen	Conjugate	Host	Isotype	Supplier	Catalogue number	Clone	Volume /1x10^6^ PBMCs
**NKp30**	BV421	Mouse	IgG1, κ	BD Biosciences, Le Pont de Claix, France	563385	p30-15	5 μL
**CD69**	ECD	Mouse	IgG2B	Beckman Coulter, Villepinte, France	6607110	TP1.55.3	5 μL
**NKG2A**	PE	Mouse	IgG2B	Beckman Coulter, Villepinte, France	IM3291U	Z199	5 μL
**CD57**	PB	Mouse	IgM	Beckman Coulter, Villepinte, France	A74779	NC1	5 μL
**Kir2DL2/3**	PE	Mouse	IgG1	Beckman Coulter, Villepinte, France	IM2278U	GL183	5 μL
**Kir3DL1**	AF700	Mouse	IgG1, κ	BioLegend, CA, USA	312712	DX9	5 μL
**NKp46**	PE-Vio615	Mouse	IgG1, κ	Miltenyi Biotec, Paris, France	130-107-456	9E2	5 μL
**NKG2C**	APC	Mouse	IgG1	RD systems, Lille, France	FAB138A-100	134591	5 μL
**NKG2D**	APC	Mouse	IgG1	Beckman Coulter, Villepinte, France	A22329	ON72	5 μL
**Siglec-7**	PE	Mouse	IgG2B	Beckman Coulter, Villepinte, France	A22330	Z176	5 μL
**PD-1**	PE	Mouse	IgG1, κ	BD Biosciences, Le Pont de Claix, France	560795	EH12.1	5 μL
**CD8**	ECD	Mouse	IgG1	Beckman Coulter, Villepinte, France	737659	SFCI21Thy2D3	5 μL
**CD16**	PerCP-Cy5.5	Mouse	IgG1, κ	BD Biosciences, Le Pont de Claix, France	560717	3G8	5 μL
**CD56**	PC7	Mouse	IgG1	Beckman Coulter, Villepinte, France	A21692	N901 (NKH-1)	5 μL
**CD3**	APC-Efluor780	Mouse	IgG1, κ	eBioscience, Courtaboeuf, France	47-0038-42	UCHT1	3 μL

### EBV peptides

Synthetic latent and lytic EBV peptides included: previously described 8–11 mers restricted to different class-I MHC molecules [25,26] (S1 Table; GeneCust Europe, Ellange, Luxembourg), previously described 11–20 mers restricted to different class-II MHC molecules [26,27] (S2 Table); Epytop, Nimes, France), 15-mers overlapping by 10 amino acids and spanning the whole BZLF-1 protein sequence (GenBank reference: AAA66529.1; S3 Table; GeneCust Europe) and 15-mers overlapping by 10 amino acids and spanning the immunogenic C-terminal region of EBNA-3A protein (GenBank reference: AFY97915.1; S4 Table; GeneCust Europe). All KTRs but one had HLA (Human leucocyte antigen) alleles matching class-I or class-II-MHC restricted optimal peptides (S5 Table).

### EBV-specific T cell detection by intracellular cytokine staining assay

PBMCs were thawed as mentioned above then suspended at 1x10^6^ cells/mL in RPMI+ medium 10% FBS and incubated overnight at 37°C, 5% CO_2_. Next day, PBMCs (0.5-1x10^6^ cells/mL) were suspended in RPMI+ medium and stimulated with BZLF-1 overlapping 15mers + class I MHC-restricted lytic-EBV optimal peptides, class I MHC-restricted latent EBV optimal peptides (2μg/mL), Staphylococcal enterotoxin B (SEB; 1μg/mL; Sigma-Aldrich) as a positive control or RPMI+ medium only as a negative control. According to sample availability, PBMCs of five patients and five controls were also stimulated with class II MHC-restricted latent EBV optimal peptides (2μg/mL). Then, brefeldin A and monensin (10μg/mL and 5μg/mL, respectively; Sigma-Aldrich) were added to PBMCs, followed by a 6 hour incubation at 37°C, 5% CO_2_. After stimulation, cells were washed with PBS1x-BSA 0.5% and suspended in 1mL of PBS 1x. All antibodies used from that moment on were from BD Biosciences (Table 3). Cells were incubated for 15 minutes with the viability stain at room temperature in the dark, then washed and stained with extracellular antibodies for 15 minutes at room temperature in the dark. The FIX & PERM^®^ Cell Permeabilization Kit was used according to manufacturer’s recommendations (Thermo Fisher Scientific). After fixation and permeabilization, cells were stained with intracellular antibodies for 15 minutes at room temperature in the dark, then washed and fixed with 300 ml of BD CellFIX^™^ 1x. Acquisition was performed in the Flow Cytometry Core CyPS (Sorbonne University, Pitie-Salpetriere Hospital, Paris, France) using a five-laser LSR Fortessa flow cytometer (BD Biosciences) standardized with the same lot of CST beads (BD Biosciences) before each acquisition.

**Table 3:** Primary antibodies used in intracellular cytokine staining assay.

Antigen	Conjugate	Host	Isotype	Catalogue number	Clone	Volume /1x10^6^ PBMCs
**CD8**	BUV395	Mouse	IgG1, κ	563795	RPA-T8	5 μL
**PD-1**	BV421	Mouse	IgG1, κ	562516	EH12.1	5 μL
**Tim-3**	BV711	Mouse	IgG1, κ	565566	7D3	5 μL
**IFN-y**	FITC	Mouse	IgG2B, κ	340449	25723.11	20 μL
**CD8**	PerCP-Cy5.5	Mouse	IgG1, κ	565310	sk1	5 μL
**IL-2**	PE-CF594	Mouse	IgG1, κ	562384	5344.111	5 μL
**TNF**	PE-Cy7	Mouse	IgG1, κ	557647	Mab11	5 μL
**CD4**	APC	Mouse	IgG1, κ	555349	RPA-T4	20 μL
**Fixable Viability Stain 700**	APC-R700			564997		1 μL
**CD3**	APC-H7	Mouse	IgG1, κ	560176	SK7	5 μL

Data were analyzed with FlowJo software version 10.5 as shown in S2A and S2B Fig. EBV-specific T cells were detected after specific stimulation by the production of IFNγ, interleukin-2 (IL-2) and tumor necrosis factor-α (TNFα) in individual gates (S2C Fig). Then, a boolean gate including the 3 cytokines (IFNγ and/or IL-2 and/or TNFα) was created to study response size and immune-checkpoint expression (S2D Fig).

Using flow cytometry, polyfunctionality was defined as the ability to co-express multiple cytokines at the single-cell level [28].To study the polyfunctionality of EBV-specific T cells, we used a Boolean combination gate strategy to create the seven possible combinations of IFNγ, IL-2 and/or TNFα cytokine production; next, data were analyzed with the Funky Cells ToolBox 0.1.2 (www.FunkyCells.com) developed by Martin Larsen, PhD. We define a T cell as polyfunctional when more than one cytokine, that is IFNγ, IL-2 and/or TNFα, is produced simultaneously. Monofunctionality is defined when only one of the studied cytokines is produced at the single-cell level; however, there are many other functional molecules that we have not studied here, that might be expressed by those cells. Thus the term “monofunctional” is restricted to the cytokines array we studied here. Data are reported after background subtraction. Polyfunctionality profiles and immune-checkpoint expression were only studied in patients with positive responses after background subtraction and at least 100 events available in the 3 cytokine Boolean gate (S2D Fig).

### ELISpot-IFNγ assay

MultiScreen HTS-IP 96-well plates (Merck Millipore, Molsheim, France) were washed three times with PBS1x, then coated with anti-human IFNγ capture antibody (1μg/mL; Mouse IgG1κ clone: B-B1; Diaclone, Besançon, France) and incubated overnight at 4°C. The next day, plates were washed and blocked with RPMI+ medium supplemented with 10% FBS for 30 minutes at 37°C, 5% CO_2_. Thawed PBMCs (1 x10^5^ cells/well) were stimulated in triplicates with RPMI+ medium supplemented with 10% FBS as negative control, phytohemagglutinin (PHA, 2μg/mL, Sigma-Aldrich) as positive control and EBV peptides (2μg/mL, then incubated at 37°C, 5% CO_2_. EBV-specific CD4^+^ T cell response was detected after 40-hour incubation with class II MHC-restricted latent EBV peptides (divided in 3pools of 11 peptides). EBV-specific CD8^+^ T cell response was detected after 20-hour incubation with BZLF-1 (divided in 5 pools of ≤10 peptides), EBNA-3A (divided in 16 pools of ≤10 peptides) and class I MHC-restricted latent (divided in 4 pools of ≤10 peptides), and lytic (1 pool of 3 peptides) EBV optimal peptides at 37°C. Plates were then washed 3 times with each PBS1x, PBS1x-Tween 20 and PBS1x. The anti-human IFN-γ detection antibody (0.5μg/mL; Mouse IgG1κ clone: B-G1; Diaclone) was added and incubated for 6 hours at 37°C. After 3 washes with PBS1x, Streptavidin-alkaline phosphatase (dilution 1:1000; GE Healthcare, France) was added and incubated for 1 hour at 37°C. After wash, 50μL/ well of NBT/ BCIP (nitro-blue tetrazolium chloride/5-bromo-4-chloro-3'-indolyphosphate p-toluidine salt; Thermo Fisher Scientific) were added and incubated at room temperature for 15 minutes in the dark. Reaction was stopped with water followed by three washes with water. Plate was air dried for at least 24 hours in the dark then Spot forming cells(SFC) were read using AID Elispot reader and AID Elispot software (Autoimmun Diagnostika, Strassberg, Germany). The mean numbers of SFC from triplicates or duplicates were adjusted to 1 x10^6^ PBMCs after background subtraction. The thawing viability threshold was set at 50% live cells, and ELISpot-IFNγ positivity threshold was 50 SFC per million cells. The mean viability by trypan blue immediately after thawing was 73% (SD = 13%) for 10 patients and 83% (SD = 19%) for 15 controls.

### Statistical analysis

Statistical analysis was performed with Graph Pad Prism version 6 for Windows (La Jolla, USA). The Mann-Whitney U test was used to assess differences between independent groups of patients. A P-value <0.05 was considered statistically significant. Correlations were determined with the Spearman rank correlation coefficient and statistical significance of P-values according to Bonferroni correction threshold (0.0041). Unsupervised hierarchical clustering was performed using R language with the heatmap.2 function from the gplots package[29] and dendrograms were generated based on the Ward.D method[30].

## Results

### Patients characteristics and lymphopenia status

Ten clinically long-term stable adult KTRs were included, with a median post-transplant duration of 12.5 years (interquartile range [IQR]: 13–25 years). At the time of blood sampling, all patients were clinically stable, and were receiving combined immunosuppressive therapy (Table 4). Six out of 10 had detectable EBV viral loads in peripheral blood. When compared to HCs, KTRs presented global lymphopenia (median: KTRs = 1,256 vs. HCs = 1,996/mm^3^; P = 0.0455; S3A Fig), involving mainly B cells (median CD19^+^: KTRs = 45 vs. HCs = 213/mm^3^, P = 0.0002; S3C Fig) and CD4^+^ T cells (median CD4^+^: KTRs = 484 vs. HCs = 1,007/mm^3^; P = 0.0007; S3E Fig). Of note, the absolute B cell and CD4^+^ T cell counts were not related to the type of immunosuppressive treatment and persisted overtime after transplantation (S3G and S3H Fig). Furthermore, follow up of CD4^+^ and CD8^+^T cell counts in 2 patients and of EBV viral loads in 5 patients, showed stable values overtime (S4 Fig). Three years after sample collection, one patient was undergoing dialysis (KTR #7), while the others were clinically stable in particular without EBV-related PTLDs.

**Table 4:** Patients characteristics.

Patient ID	Sex	Indication for transplantation	Age at transplant	Induction therapy	Immunosuppressive therapy			Time post-transplant (years)	Viral load (PCR)		
					Glucocorticoids	Calcineurin inhibitors	Antimetabolites		EBV^a^	CMV^a^	HHV-8^b^
**KTR 1**	M	IgA Glomerulonephropathy	53	Basiliximab	+	+	+	9	3.72	<1.4	n/a
**KTR 2**	M	Glomerulonephropathy	30	None	+	+	-	37	2.81	n/a	<10
**KTR 3**	M	Diabetic Glomerulonephropathy	43	ATG (7 days)	+	-	+	22	2.72	<1.4	<10
**KTR 4**	M	Accidental severe organ damage	39	ATG (7 days)	+	+	+	12	3.25	<1.4	<10
**KTR 5**	F	Glomerulonephropathy	32	ATG (7 days)	+	+	+	3	<1.4	<1.4	<10
**KTR 6**	F	Glomerulonephropathy	41	ATG (21 days)	+	-	+	18	4.03	2.4	n/a
**KTR 7**	M	Diabetic Glomerulonephropathy	57	ATG (6 days)	+	+	+	2	2.91	<1.4	<10
**KTR 8**	M	IgA Glomerulonephropathy	25	ATG (14 days)	+	+	+	17	<1.4	n/a	<10
**KTR 9**	F	Renal polycystosis	41	ATG (14 days)	+	+	+	13	<1.4	<1.4	<10
**KTR 10**	M	Renal polycystosis	71	Basiliximab	+	+	+	2	<1.4	1.5	<10
**Median**			41					12.5	3.1		

^a^Log copies/ml

^b^Copies/10^6^ cells. PCR, polymerase chain reaction; EBV, Epstein-Barr virus; CMV, cytomegalovirus; HHV-8, human herpesvirus 8; n/a, not available.

### Low Th1 CD4^+^ T cells against latent EBV antigens in long-term stable kidney transplant recipients

**Fig 1:**
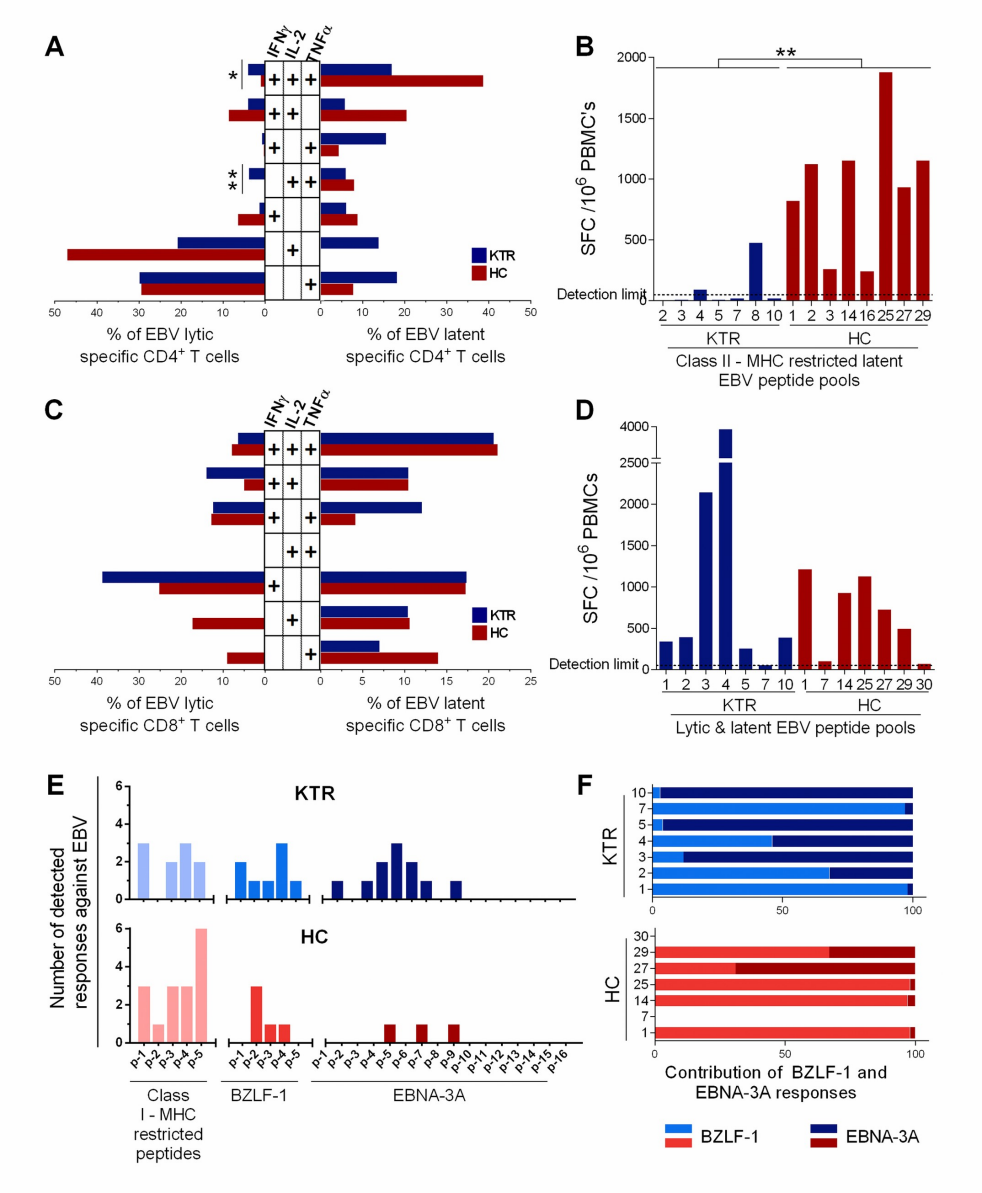
EBV-specific T cell responses in long-term kidney transplant recipients (KTRs) and healthy controls (HCs). (A) Polyfunctionality profile of latent (responders, KTRs n = 3/5; HCs n = 4/5) and lytic (responders, KTRs n = 8/10; HCs n = 13/15) EBV-specific CD4^+^ T cells from responding KTRs and HCs, displaying each possible combination of cytokine (IFNγ, IL-2, TNFα) production, generated by Boolean gate strategy. (B) Latent EBV-specific CD4^+^ T cell response measured by ELISpot-IFNγ as spot forming cells (SFC)/10^6^ PBMC. (C) Polyfunctionality profile of latent (responders, KTRs n = 9/10; HCs n = 11/15) and lytic (responders, KTRs n = 9/10; HCs n = 14/15) EBV-specific CD8^+^ T cells from responding KTRs and HCs. (D) EBV-specific CD8^+^ T cell response against latent and lytic EBV peptides measured by ELISpot-IFNγ as SFC/10^6^ PBMC. (E) Diversity of ELISpot-IFNγ responses against each peptide pool by group. (F) Proportion of BZLF-1 vs. EBNA-3A T cell responses within the total sum of both responses by ELISpot-IFNγ (SFC/10^6^ PBMCs). Exact P-values were calculated with a two-tailed Mann-Whitney test; only significant values (P<0.05) are shown.

We studied the EBV-specific CD4^+^T cell response directed against lytic and latent EBV proteins. According to PBMC availability, we used overlapping 15 mer-peptides spanning the whole lytic BZLF-1 protein (KTRs n = 10, HCs n = 15), and class II MHC-restricted latent EBV optimal peptides (KTRs n = 5, HCs n = 5). Intracellular cytokine staining (ICS) assays identified CD4^+^ and CD8^+^ T specific responses by multiparameter flow cytometry (S2 Fig). The frequencies of CD4^+^ T cells producing IFNγ, IL-2, or TNFα against latent and lytic EBV peptides were similar in KTRs and HCs although the latent–specific responses were particularly weak in both groups (S5A Fig). Next, we characterized the functional capacity and immunecheckpoint expression of those EBV-specific cells from responding KTRs and HCs. The polyfunctionality profile showed that the lytic specific CD4^+^ T cells were monofunctional in responders of both groups (responders, KTRs n = 8/10; HCs n = 13/15) suggesting an effector memory CD4^+^ T cell response, while the latent specific CD4^+^ T cells were mainly polyfunctional suggesting a central memory CD4^+^ T cell response (responders, KTRs n = 3/5; HCs n = 4/5) (Fig 1A). There was no difference in PD-1 and Tim-3 expression by latent and lytic EBV-specific CD4^+^ T cells between groups (S5B–S5D Fig). Of note, the number of events detected in the CD4^+^IFNg^+^ gate was two- to ten-fold higher than in the CD8^+^IFNg^+^ gate confirming that the class II MHC-restricted EBV optimal peptides we used preferentially triggered CD4^+^ T cells (S6 Fig). Considering the weak latent–specific responses, we further studied the CD4^+^ T cell responses against class II MHC-restricted latent-EBV optimal peptides with an ELISpot-IFNγ assay, a more sensitive technique than ICS assay that detect Th1 effector CD4^+^ T cell responses. We found that the latent EBV-specific MHC Class II restricted T cell responses were significantly lower in KTRs when compared to HCs (2/7 responders vs. 8/8 responders; median SFC/10^6^ PBMC: KTRs = 24 vs. HCs = 1032; P = 0.0012; Fig 1B). This was not due to an immunodominance bias because the HLA-typing of KTRs was diverse and adapted to MHC-class II restricted peptides (S5 Table).

Together, those results show that in KTRs CD4^+^T cell responses against latent EBV proteins are weak while the EBV lytic responses are conserved. They also show that the lytic EBV-specific CD4^+^ T cell responses are monofunctional suggesting an effector and effector memory profile, while the latent EBV-specific CD4^+^ T cell responses are polyfunctional suggesting a central memory phenotype. The loss of the Th1 effector/effector memory latent specific CD4^+^ T cells is in line with the total CD4^+^ T cell lymphopenia observed in KTRs (S3E Fig), and evokes a new mechanism for predisposition to EBV-related complications in this immunosuppressed population.

### Broader diversity of CD8^+^ T cell responses against EBNA-3A in KTRs

We next studied the EBV-specific CD8^+^T cells directed against lytic and latent EBV proteins using BZLF-1 overlapping 15 mers and class I MHC-restricted lytic and latent EBV optimal peptides by ICS. The lytic and latent EBV-specific CD8^+^ T cell responses were similar between KTRs and HCs in terms of frequency (EBV lytic responders: 9/10 KTRs vs. 14/15 HCs; EBV latent responders: 9/10 KTRs vs. 11/15 HCs; S5E Fig). The cytokine-secretion profile of lytic EBV-specific CD8^+^ T cells was mainly monofunctional, with higher frequencies of IFNγ^+^ IL-2^-^ TNFα^-^ specific cells in KTRs when compared to HC group, suggesting an effector-differentiation (P = 0.0831; Fig 1C). The latent EBV-specific CD8^+^ T cells showed similar cytokine production profiles in KTRs and HCs, with equal distribution between polyfunctional and monofunctional cells (Fig 1C). Moreover, PD-1 expression by EBV-specific CD8^+^ T cells was similar between groups (S5F and S5H Fig) and was not correlated with the EBV viral load (S7 Fig).

We further studied the diversity of the CD8^+^ T cell responses against EBV immunodominant proteins lytic BZLF-1, and latent EBNA-3A, by ELISpot-IFNγ assay. We tested in parallel the class I-MHC restricted latent and lytic EBV optimal peptides. Of note, the CD8^+^T cell responses to those antigens detected with ICS by flow cytometry (S5E Fig) were approximately 10 times greater than the CD4^+^ T cell ones (S5A Fig), thus we considered that responses detected against BZLF-1 and EBNA-3A 15-mer peptides by ELISpot-IFNγ assay were mainly of CD8^+^ T cell origin.

All KTRs (n = 7/7) and HCs (n = 7/7) responded against at least one EBV peptide pool in ELISpot assay, with a wide range of reactivity observed between individuals; although the total (latent and lytic) EBV-specific CD8^+^ T cell responses, measured as SFC x10^6^ PBMCs, showed similar median values between groups (median: KTRs = 390 vs. HCs = 733; P = 0.7991; Fig 1D). However, the BZLF-1-specific-T cells of KTRs were directed within all five peptides pools, while the responses of HCs were directed at pools 2 to 4 corresponding to the central region of the BZLF-1 protein (responders KTRs n = 5/7, HCs 5/7; Fig 1E). Interestingly, the KTRs EBNA-3A responses were mainly directed against the N-terminal portion of the protein (AA position 133 to 542), and only KTRs responded to EBNA-3A peptide pools 1 to 4, containing the EBNA-3 family homology domain[31] (Fig 1E), contrasting with the HCs responses which were directed towards the central region of the EBNA-3A sequence (EBNA-3A responders KTRs n = 5/7, HCs 2/7). Thus, when taking in count the proportional contribution of those responses against EBNA-3A or BZLF-1 only, the KTRs CD8^+^ T cell responses seem skewed to the N-terminal portion of the EBNA-3A protein (Fig 1F).

Together, those results, although observed in a little sample of patients, suggest that effector CD8^+^ T cell responses against EBV diversify after kidney transplantation, possibly because of active EBV infection. Particularly, the EBNA-specific CD8 T cell response in KTRs may diversify because B cells establish a transcription program in type III latency cycle, a program were all EBV-EBNA proteins are expressed.[32]

### Alterations of EBV-specific effector T cell responses segregate KTRs from HCs

**Fig 2:**
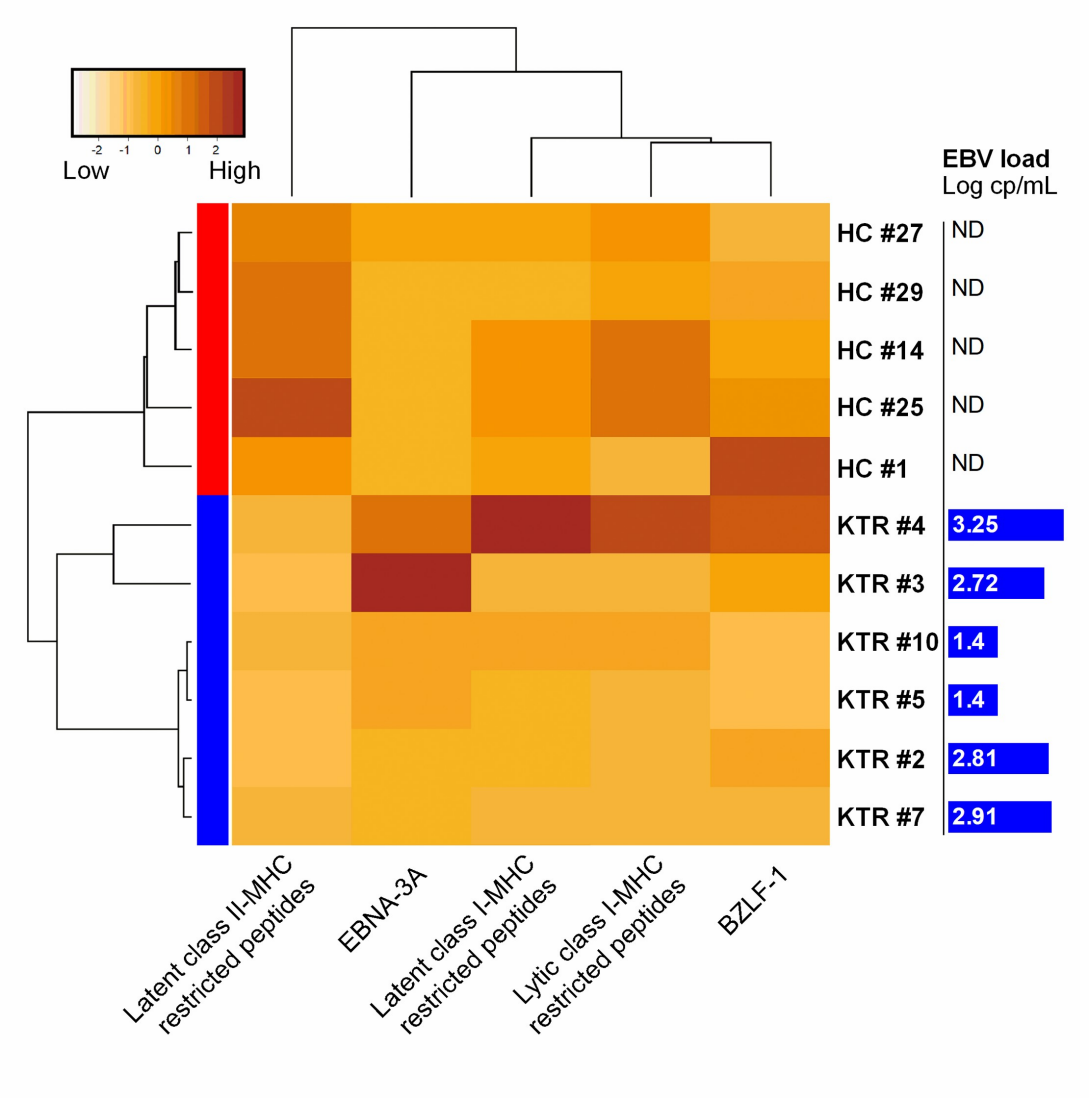
Hierarchical clustering of kidney transplant recipients (KTRs) and healthy controls (HCs) according to effector T cell responses. Heatmap plot of EBV-specific CD4^+^ Th1 and CD8^+^ effector T cell responses detected by ELISpot-IFNγ. Lines are individual KTRs (Blue) or HCs (Red). Columns are each peptide pool tested separately in ELISpot IFNg assay. Color scale represents the magnitude of the response expressed in spot forming cells (SFC)/10^6^ PBMCs. Dendrograms were generated using unsupervised hierarchical clustering based on the Ward.D method. Last column to the right show the EBV load determined at simultaneous blood sample in Log copies/mL.

Because our results showed several modifications of EBV-specific effector/effector memory T cells in KTRs, we studied in KTRs and HCs those EBV-specific responses with unsupervised hierarchical clustering in order to demonstrate that the EBV-specific T cell immune profile is different in KTRs than in HC. We selected 5 HCs and 6 KTRs from whom we had all available ELISpot-IFNγ assays. As shown in Fig 2, KTRs clustered separately from HCs when taken into account both latent and lytic EBV-specific effector/effector memory CD4^+^ and CD8^+^ T cell responses. Such segregation was mainly influenced by the latent CD4^+^ T cell responses and the EBNA-3A-specific CD8^+^ T cell responses. Interestingly, we observed 2 clusters within the KTR group: the closest cluster to HCs was composed by two patients who presented strong responses against both EBNA-3A and BZLF-1 proteins and high EBV loads, while the other four KTRs had overall similar responses and presented mild or undetectable viral loads (Fig 2).

Taken together, those data suggest that the modifications of the EBV-specific T cell responses in KTRs might reflect the balance between the enhancement of cellular immunity induced by persistent viral antigenic stimulation (high EBV viral loads), and the life-long immunosuppression that impacts mainly the CD4^+^ T cell compartment (S3E Fig). The resulting EBV-specific immunity is a skewed CD8^+^ T cell response towards EBNA-3A while the latent CD4^+^ specific-T cells remain barely detectable.

### Peripheral blood NK cells characteristics in long-term kidney transplant recipients

**Fig 3:**
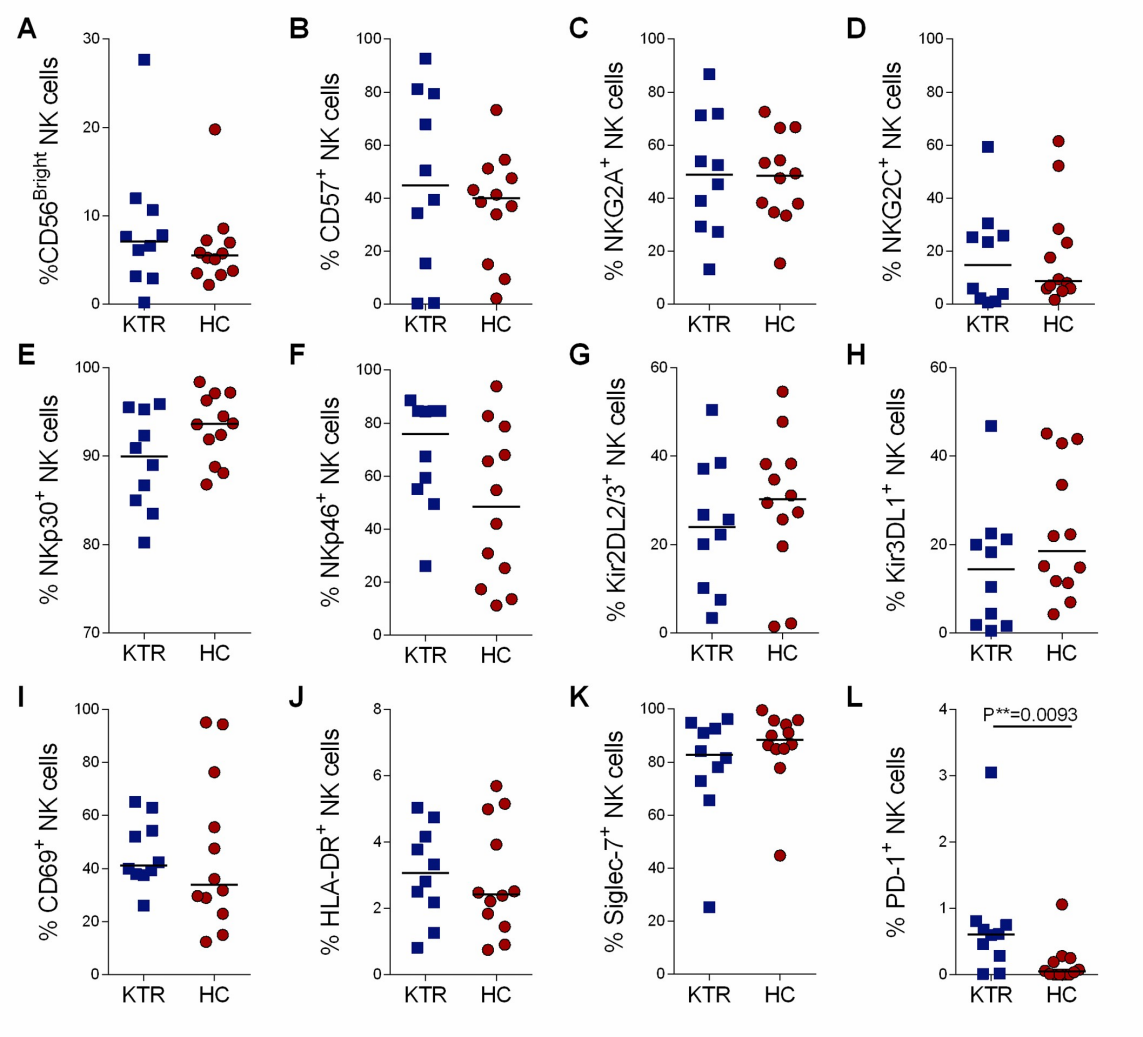
Phenotypic features of NK cells in kidney transplant recipients (KTRs) and healthy controls (HCs). Percentage of A) CD56^Bright^, B) CD57^+^, C) NKG2A^+^, D) NKG2C^+^, E) NKp30^+^, F) NKp46^+^, G) Kir2DL2/3^+^, H) Kir3DL1^+^, I) CD69^+^, J) HLA-DR^+^, K) Siglec-7^+^ and L) PD-1^+^ in CD3^-^CD56^+^ NK cells from 10 kidney transplant recipients (KTRs) and 12 healthy controls (HCs). Expression was measured at the surface by flow cytometry on thawed PBMCs. Horizontal bars indicate the median. Exact P-values were calculated with a two-tailed Mann-Whitney test.

NK cells are the first line of cellular defense against EBV infection, thought it has been poorly described in KTRs. We thus performed a detailed phenotype of peripheral NK cells in 10 KTRs and 12 HCs. First, both groups presented similar proportions of CD56 ^Bright^ and CD56^dim^ subsets (Fig 3A, S8A Fig). NK cells from KTRs were indistinguishable from those of HCs in terms of cell-surface expression of the main NK receptors studied, including c-lectin receptors (NKG2C and NKG2A), natural cytotoxicity receptors (NKp30 and NKp46), and killer immunoglobulin receptors (Kir2DL2/3 and Kir3DL1), along with other tested activation and NK cell markers, such as HLA-DR, CD69, CD57, and Siglec-7 (Fig 3B–3K). In contrast, the frequency of PD-1^+^ NK cells, though low, was significantly higher in KTRs than in HCs (median %: KTRs = 0.61 vs. HCs = 0.05; P = 0.0093; Fig 3L) despite similar PD-1 mean fluorescence intensity (MFI) between groups (S8B Fig). Thus, these data suggest that both the chronic allo-antigenic stimulation and the immunosuppressive regimens used in long-term kidney transplantation have no major impact on the NK cell compartment, except for a slight PD-1 over-expression which could alter NK cell-mediated cytotoxicity and cytokine production.

### PD-1 normalizes over time on kidney transplant recipients CD8^+^ T cells

**Fig 4:**
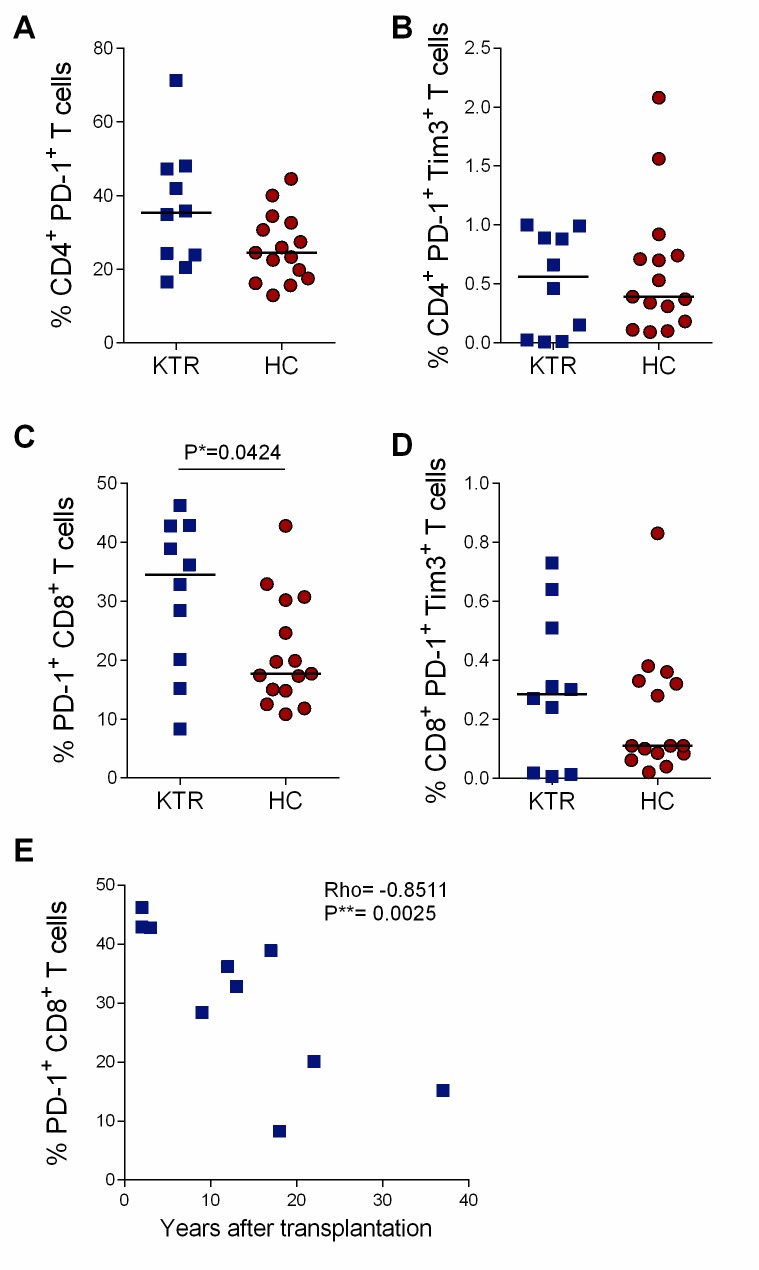
T cell activation/exhaustion phenotype during kidney transplantation. PD-1 and Tim-3 expression on A) CD4^+^ and B) CD8^+^ peripheral blood total T cells. Data are shown for 10 kidney transplant recipients (KTRs) and 15 healthy controls (HCs). C) Correlation between the frequency of PD-1^+^ CD8^+^ T cells and the number of years after transplantation in 10 KTRs. Horizontal bars indicate the median. Exact P-values were calculated with a two-tailed Mann-Whitney test and the correlation was assessed with the Spearman rank correlation coefficient. Bonferroni significativity threshold for correlations was 0.0041.

We next examined whether the expression of the PD-1 immune checkpoint molecule was also increased on CD4^+^ and CD8^+^ T cells, as observed on NK cells. On total CD4^+^ T cells, the activation and exhaustion phenotype showed similar PD-1 expression and PD-1/Tim-3 co-expression in KTRs and HCs (Fig 4A and 4B), while the expression of the pre-apoptotic marker CD95 was increased in KTRs when compared to HCs (median: KTRs = 73% vs. HCs = 51%; P = 0.0003; S9A Fig). Conversely, the proportions of PD-1^+^ CD8^+^ T cells were two-fold higher in KTRs, compared to HCs (median %: KTRs = 34.5 vs. HCs = 17.7; P = 0.0424; Fig 4C). Interestingly, the proportion of PD-1^+^CD8^+^ T cells decreased with time with a significant negative correlation with post-transplantation duration (P = 0.0025, Rho = -0.8511; Fig 4E). Moreover, when compared to HCs, total CD8^+^ T cells of KTRs were significantly activated (median HLA-DR^+^ CD8^+^ cells, KTRs = 37% vs. HCs = 13%, P = 0.0003; median HLA-DR^+^ CD38^+^ CD8^+^ T cells, KTRs = 9% vs. HCs = 4%, P = 0.0005; median CD95^+^ CD8^+^ T cells, KTRs = 47% vs. HCs = 38%, P = 0.0356; S9B Fig). Of note, none of those activation markers diminished with time after transplantation or was correlated with EBV loads (S6 Table), indicating that this persistent activation probably results from homeostatic activation due to immunosuppression. The PD-1 overexpression by CD8^+^ T cells which normalizes with time after transplantation suggests that the allogeneic chronic stimulation fade overtime and that peripheral immune tolerance might be gradually established.

In addition to the activation markers expression, the analysis of T cell differentiation patterns in KTRs showed a shift from naïve to effector-memory CD4^+^ T cells proportions, when compared to HCs (median naïve, KTRs = 18% vs. HCs = 34%; P = 0.0029; median effector-memory, KTRs = 58% vs. HCs = 37%, P = 0.0029; S10A Fig). Similar results were obtained for CD8^+^ T cells, with significantly lower proportions of naïve (median: KTRs = 12% vs. HCs = 37%, P<0.0001) and central-memory cells (median: KTRs = 0.7% vs. HCs = 3.0%, P<0.0001) and higher proportions of terminally differentiated effector memory CD8^+^T cells (median: KTRs = 63% vs. HCs = 19%, P<0.0001; S10B Fig), in KTRs when compared to HCs. Of note, terminally differentiated effector memory T cells (TEMRA), defined as CCR7^-^CD45RA^+^, are known to be mainly CD57^+^.^.^[33] In agreement, senescent CD57^+^ CD8^+^ T cells were also increased in KTRs (median: KTRs = 37% vs. HCs = 14%, P = 0.0004; S10B Fig). No relation was observed between those alterations and the time after transplantation or EBV viral loads (S6 Table), suggesting that the CD4^+^ and CD8^+^ T cell differentiation towards a senescent phenotype persists long-term after kidney transplantation, independently of EBV replication.

## Discussion

Our characterization of the EBV-specific T cell responses in long-term immunosuppressed but clinically stable KTRs showed specific alterations with a decrease of peripheral CD4^+^ T cell immunity against latent EBV antigens and modifications of the EBV-specific CD8^+^ T cell responses that were directed against the N-terminal portion of the EBNA proteins. Those modifications were associated with total T and NK cell phenotypic alterations.

To our knowledge, this is the first time that low circulating Th1 effector CD4^+^ T cells against latent EBV antigens have been reported in clinically-stable adult KTRs. Because in our study the CD4^+^ T cell responses against lytic EBV peptides were similar in KTRs and in HCs, the specific loss of CD4^+^ T cell responses against latent EBV peptides cannot be fully explained by the persistent CD4^+^ T cell lymphopenia observed in our patients. Polymorphisms of the EBNA-1 sequence have been reported in EBV+PTLD patients and healthy controls who have detectable CD8^+^ T cell responses against the viral variant but not against the prototype EBV laboratory strain B95-8[34]; thus, protein sequence variations might explain low detection of specific-CD4^+^ T cells, except the class II MHC-restricted peptide pools we used were composed of 6 different latent EBV proteins and all of tested HCs strongly recognized at least one peptide pool. However, we had no access to the HLA alleles of HCs and could not exclude if the absence of T cell responses against class II MHC-restricted peptides was linked to an immunodominance bias. Nevertheless, all but one KTR tested had class-II MHC alleles that fitted with the MHC class-II peptide list used in the ELISpot-IFNγ assay. Such a lack of latent EBV-specific CD4^+^ T cells has been identified in various types of non-Hodgkin lymphoma (NHL) in human immunodeficiency virus (HIV)-patients[35] and in immunocompetent individuals with EBV-related lymphoma,[36] as well as in one transplant patient with EBV-related PTLD.[37] In addition, prolonged EBV-seronegativity has been observed in solid organ transplant recipients who develop early-onset EBV+PTLDs.[38] Altogether, these data suggest that defective EBV-specific CD4^+^ T cell immunity could favor NHL development and particularly PTLDs.

In contrast, we also found that KTRs had similar proportions of EBV-specific CD8^+^ T cell responses compared to HCs, regardless of detectable EBV loads in blood of most patients, which reflects active EBV-infection. However, fewer HCs had responses against EBNA-3A protein while KTRs had a broader spanning of T cell responses against BZLF-1 and EBNA-3A proteins than HCs. One likely hypothesis is that active EBV infection exposes KTRs to a broader repertory of EBV epitopes while the expansion of those specific CD8^+^ T cells is impacted by life-long immunosuppressive treatment; consequently, such balance is traduced in conserved proportions of circulating EBV-specific CD8^+^ T cells in KTRs, when compared to HCs. In agreement, unsupervised hierarchical clustering analysis clearly segregated controls from patients according to EBV-specific effector T cell responses. Furthermore, the broader spanning of T cell responses against EBNA-3A protein in KTRs might reflect the abundance of EBV latency III-infected B cell, yet, none of the KTRs studied here-in however had developed any EBV-related complication. Thus, those results raise the question whether diversity and repertory of T cell responses against latency III EBV proteins have a protective role in adult KTRs against EBV-related post-transplant diseases, which could favor the development of new immunotherapies. Indeed, the main goal of prophylactic and therapeutic immunotherapies against EBV-related complications is to provide or induce protective T cell responses able to eliminate EBV-infected tumor cells expressing viral proteins [39]. First, the definition of EBV epitopes matching different HLA molecules are fundamental for the development of “off-the-shelf” EBV-specific CTLs for adoptive transfer. [40] Second we provide here the EBV-specific T cell repertoire of a group of PTLD-free patients, evoking an immune-correlate of tumor control. The recognition of specific regions of EBNA-3A protein we observed could thus be interesting, if confirmed, for the development of vaccination and CTL adoptive transfer strategies; especially taking in count that EBV-related lymphomas of transplant recipients often express latency III-infection program [2], which includes the EBNA-3 family proteins [32].

In parallel, we examined the T cell peripheral blood phenotype of KTRs. We observed a switch from naïve to terminally differentiated effector memory CD8^+^ T cell populations, characteristic of immunosenescence observed in the elderly[41] but also in KTRs.[8,42,43] Importantly, we also showed a persistent CD8^+^ T cell activation beyond 12 years after kidney transplantation which to our knowledge, has been described early after transplantation in the context of chronic viral infections[13,44] graft rejection, [10,45] and cancers but not in clinically-stable transplant recipients.[46] Noteworthy the increased PD-1 expression on CD8^+^ T cells normalized with time, suggesting that the allogeneic stimulation decreases overtime and thus that peripheral tolerance might be gradually established with time after kidney transplantation. Moreover, such a discordance with increased activation markers and senescent cells could be linked with persistent low-grade viral replication (EBV, but also CMV) or T cell lymphopenia and homeostatic activation. In agreement with our results, Moran et al. observed increased PD-1 expression by total CD8^+^ T cells of pediatric renal transplant recipients, compared to pre-transplantation and regardless of EBV loads patients carried; the mean time from transplantation to the study was 3.3 years. [15] Thus, those findings comply with our hypothesis that PD-1 expression increases early after transplantation, independently of EBV loads. Interestingly, another study of adult kidney transplant recipients immunophenotype have shown an association between increased PD-1 expression by CD8^+^ T cells and increased risk of graft rejection [47], authors of that study conclude that increased PD-1 expression might result from chronic immuneactivation; which is also in line with our hypothesis of chronic allogeneic stimulation.

Our results also show an increased expression of PD-1 by NK cells of KTRs though at low levels. Such a PD-1 overexpression by NK cells has recently become of interest in the context of persistent CMV infection and cancer, [23,48–51]. Nevertheless further confirmation is required to clearly determine whether PD-1 expression by NK cells, in addition to PD-1 overexpression by CD8^+^ T cells, could compromise the main antiviral and antitumor NK cell functions in KTRs.

The low number of subjects included and the retrospective design of our study are two main limitations. Further prospective studies with larger cohorts and longer follow-up, particularly including patients who will develop further EBV-related PTLD, are thus required to highlight whether the immune alterations described here-in are protective or detrimental and if they allow to identify patients at risk for late complications. Although we were limited to explore the regulatory T cells in our patients, there is growing evidence of the role of induced graft immune tolerance by regulatory T cells in kidney transplant recipients [52,53]. Therefore, it would have been interesting to explore the fluctuations of regulatory T cell populations in a group of kidney transplant recipients with a long post-transplant period without rejection events.

In conclusion, here we show for the first time that all immunomodulating agents after renal transplantation namely immunosuppressive drugs, alloantigenic stimulation and chronic EBV infection results in modifications of the EBV-specific T cell repertoire with skewed responses towards the N-terminal portion of the EBNA-3A protein and with defective EBV-specific Th1 effector CD4^+^ T cell responses. These findings provide new insights in the immunopathology of EBV infection after renal transplantation that could help to identify immune biomarkers for predicting EBV-related PTLD diagnosis, and to develop new preventive immunotherapies against EBV.

## Supporting information

S1 Fig Example of gating strategy used for the NK cell phenotype by flow cytometry. Multiparametric flow cytometry data were analyzed with FlowJo software. After doublets exclusion in Forward scatter height (FSH-H) versus area (FSH-A), lymphocytes were selected with Side scatter area (SSC-A) versus Forward scatter area (FSH-A). NK cells were selected as CD3^-^CD56^+^ lymphocytes. Expression of c-lectin receptors (NKG2C and NKG2A), natural cytotoxicity receptors (NKp30 and NKp46), killer immunoglobulin receptors (Kir2DL2/3 and Kir3DL1) and activation and NK cell markers (HLA-DR, CD69, CD57, and Siglec-7 and PD-1) was analyzed on CD3^-^CD56^+^ lymphocytes. Each marker was gated to measure its expression in CD56^bright^ and CD56^dim^ cell-subsets. PD-1 fluorescence minus one (FMO) staining technique was used to determine PD-1 positivity detection limit.
(TIF)

S2 Fig Example of the gating strategy used to detect EBV-specific T cells by flow cytometry with intracellular cytokine staining. (A) Live CD3^+^ T cells were selected within total lymphocytes after doublet exclusion. (B) PD-1 and Tim-3 expression by total CD4^+^ and CD8^+^ T cells was measured under unstimulated condition according to FMO controls. (C) EBV-specific T cells were detected by cytokine production (IFNγ, IL-2, TNFα) out of total CD4^+^ and CD8^+^ T cells and pooled in a (D) single boolean gate to measure PD-1 and Tim-3 expression and co-expression.
(TIF)

S3 Fig Lymphocyte subpopulations in long-term kidney transplant recipients (KTRs) and healthy controls (HCs). Absolute numbers of: (A) CD45^+^ lymphocytes, (B) CD3^-^CD56^+^/CD16^+^ NK cells, (C) CD19^+^ B cells; (D) CD3^+^ T cells; (E) CD4^+^ and CD8^+^ T cells; and (F) CD4/CD8 ratio from 10 kidney transplant recipients (KTRs) and 30 healthy controls(HCs). Horizontal bars indicate the median. Correlations between absolute counts of (G) CD19^+^ or (H) CD4^+^ T lymphocytes and the number of years after transplantation in 10 KTRs. Exact P-values were calculated with a two-tailed Mann-Whitney test and correlation was assessed with the Spearman rank correlation coefficient. Bonferroni significativity threshold for correlations was 0.0041.
(TIF)

S4 Fig Absolute T cell counts and EBV load follow up after kidney transplantation. Absolute numbers of CD4^+^ and CD8^+^ T cells and EBV loads (Log copies/mL) at different time points in two kidney transplant recipients (KTRs): (A) KTR #9 and (B) KTR #10. EBV loads (Log copies/mL) at different time points in (C) KTR #1, (D) KTR #6 and (D) KTR #7. Dotted lines indicate the blood sample in this study. EBV load detection limit is 1.4 log copies/mL.
(TIF)

S5 Fig Frequency and Immune-checkpoint expression of EBV-specific T cells in kidney transplant recipients (KTRs). (A) Frequency of latent (KTRs n = 5; HCs n = 5) and lytic (KTRs n = 10; HCs n = 15) EBV-specific CD4^+^ T cells (IFNγ, IL-2, and TNFα) determined by intracellular cytokine staining assay with flow cytometry. Frequency of (B) PD-1 and (C) Tim-3 expression and (D) co-expression on latent (responders: KTRs n = 3/5; HCs n = 4/5) and lytic (responders: KTRs n = 8/10; HCs n = 13/15) EBV-specific CD4^+^ T cells from responding KTRs and HCs. (E) Frequency of latent and lytic EBV-specific CD8^+^ T cells (KTRs n = 10; HCs n = 15). Frequency of (F) PD-1 and (G) Tim-3 expression and (H) co-expression on latent (responders: KTRs n = 9/10; HCs n = 11/15) and lytic (responders: KTRs n = 9/10; HCs n = 14/15) EBV-specific CD8^+^ T cells from responding KTRs and HCs. Horizontal bars indicate the median. Exact P-values were calculated with a two-tailed Mann-Whitney test; only significant values (P<0.05) are shown.
(TIF)

S6 Fig Comparison of IFNγ production by CD4^+^ and CD8^+^ T cells after stimulation with latent EBV class II MHC-restricted peptides. PBMCs of one kidney transplant recipient (KTR 8) and one healthy control (HC 2), were stimulated with media or with latent EBV class II MHC-restricted peptides to verify the specificity of peptide recognition. Dot plot shows IFNγ^+^ cells within total CD4^+^ or CD8^+^ T cells detected by intracellular cytokine (IFNγ) staining assay in flow cytometry.
(TIF)

S7 Fig Association between PD-1 expression by EBV-specific CD8^+^ T cells and EBV viral load in kidney transplant recipients (KTRs). The correlation between the frequency of PD-1 expression on lytic-EBV-specific CD8^+^ T cells from responding KTRs (n = 9/10) and the EBV load (Log of copies/mL) was assessed with the Spearman rank correlation coefficient. Bonferroni significativity threshold was 0.0041.
(TIF)

S8 Fig Supplemental phenotypic features of NK cells in kidney transplant recipients (KTRs) and healthy controls (HCs). Percentage of (A) CD56^Dim^ CD3^-^ NK cells and (B) mean fluorescence intensity (MFI) of PD-1 in total CD56^+^CD3^-^ NK cells from 10 kidney transplant recipients (KTRs) and 12 healthy controls (HCs). Expression was measured at the surface by flow cytometry on thawed PBMCs. Horizontal bars indicate the median. Exact P-values were calculated with a two-tailed Mann-Whitney test.
(TIF)

S9 Fig T cell activation phenotype during kidney transplantation. Frequency of (A) CD4^+^ and (B) CD8^+^ T cells expressing common activation markers detected by flow cytometry. Data are shown for kidney transplant recipients (KTRs; n = 10) and healthy controls (HCs; n = 30). Horizontal bars indicate the median. Exact P-values were calculated with a two-tailed Mann-Whitney test.
(TIF)

S10 Fig T cell differentiation during kidney transplantation. Frequency of (A) CD4^+^ and (B) CD8^+^ T cell differentiation subtypes detected by flow cytometry. Data are shown for kidney transplant recipients (KTRs; n = 10) and healthy controls (HCs; n = 30). Horizontal bars indicate the median. Exact P-values were calculated with a two-tailed Mann-Whitney test.
(TIF)

S1 Table Sequences of 42 class I MHC-restricted latent and lytic EBV optimal peptides. (PDF)

S2 Table Sequences of 33 class II MHC-restricted latent EBV optimal peptides. (PDF)

S3 Table Sequences of BZLF-1 overlapping peptides. 47 15mer peptides overlapping by 10 amino acids and covering the lytic BZLF-1 protein.
(PDF)

S4 Table Sequences of EBNA-3A overlapping peptides. 160 15mer peptides overlapping by 10 amino acids and partially covering the latent EBNA-3A protein.
(PDF)

S5 Table Class I- and class II-HLA alleles and predicted responses to EBV optimal peptides of kidney transplant recipients (KTRs). ■ Predicted and positive response; 〼Predicted and negative response; ●Unpredicted and positive response; □Predicted but undetermined response.
(PDF)

S6 Table Correlations between T cell phenotype, time after transplantation and EBV load of kidney transplant recipients. EBV, Epstein-Barr virus. ^a^Bonferroni significativity threshold ≤ 0.0041
(PDF)
