# Supplementary figures and images for: Skewed T cell responses to Epstein-Barr virus in long-term asymptomatic kidney transplant recipients
Source: PLoS One. 2019 Oct 22;14(10):e0224211. doi: 10.1371/journal.pone.0224211 (PMC6804993; doi:10.1371/journal.pone.0224211)

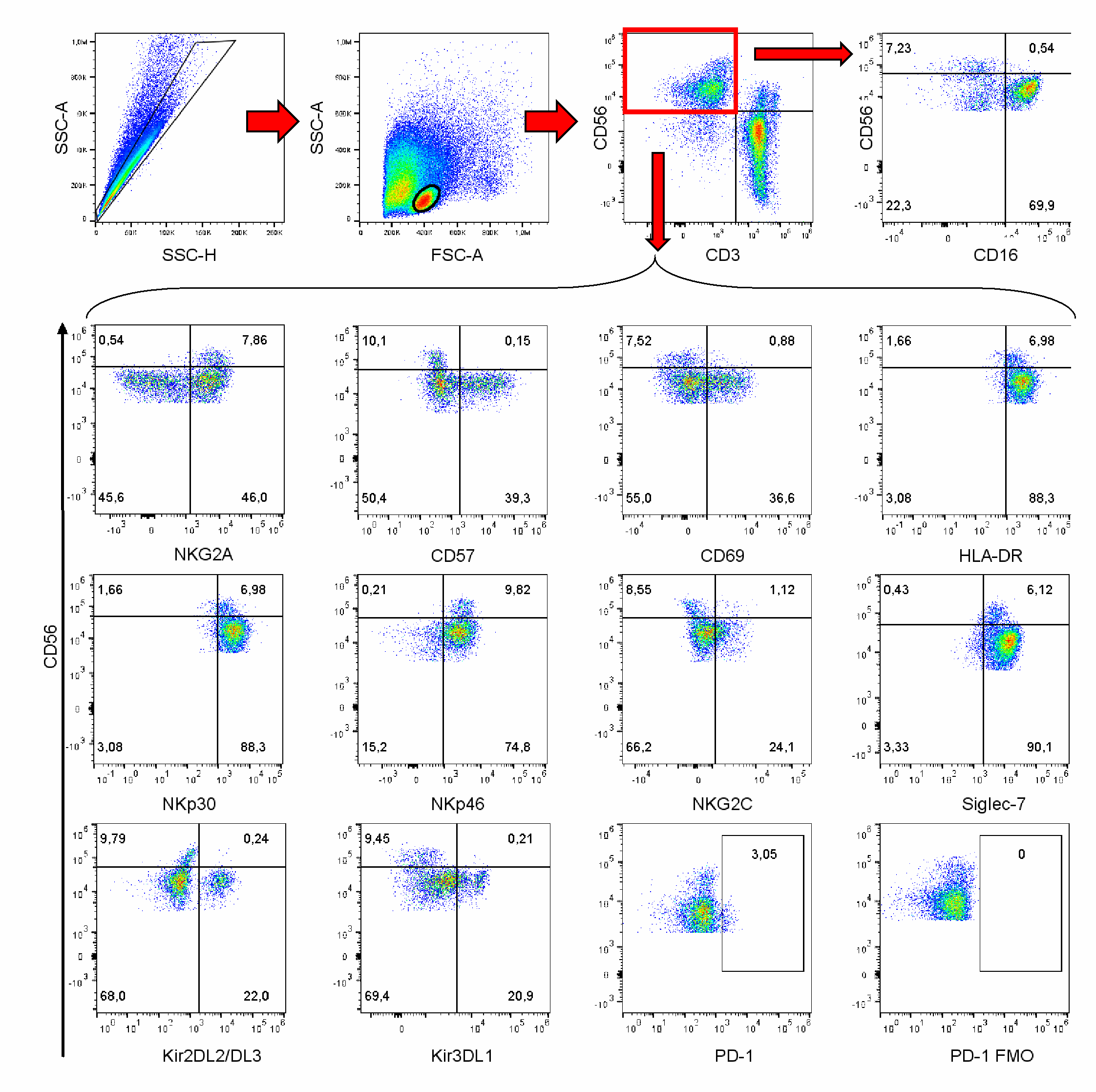

Supplement: S1 Fig — Multiparametric flow cytometry data were analyzed with FlowJo software. After doublets exclusion in Forward scatter height (FSH-H) versus area (FSH-A), lymphocytes were selected with Side scatter area (SSC-A) versus Forward scatter area (FSH-A). NK cells were selected as CD3-CD56+ lymphocytes. Expression of c-lectin receptors (NKG2C and NKG2A), natural cytotoxicity receptors (NKp30 and NKp46), killer immunoglobulin receptors (Kir2DL2/3 and Kir3DL1) and activation and NK cell markers (HLA-DR, CD69, CD57, and Siglec-7 and PD-1) was analyzed on CD3-CD56+ lymphocytes. Each marker was gated to measure its expression in CD56bright and CD56dim cell-subsets. PD-1 fluorescence minus one (FMO) staining technique was used to determine PD-1 positivity detection limit. (TIF) [file pone.0224211.s001.tif]

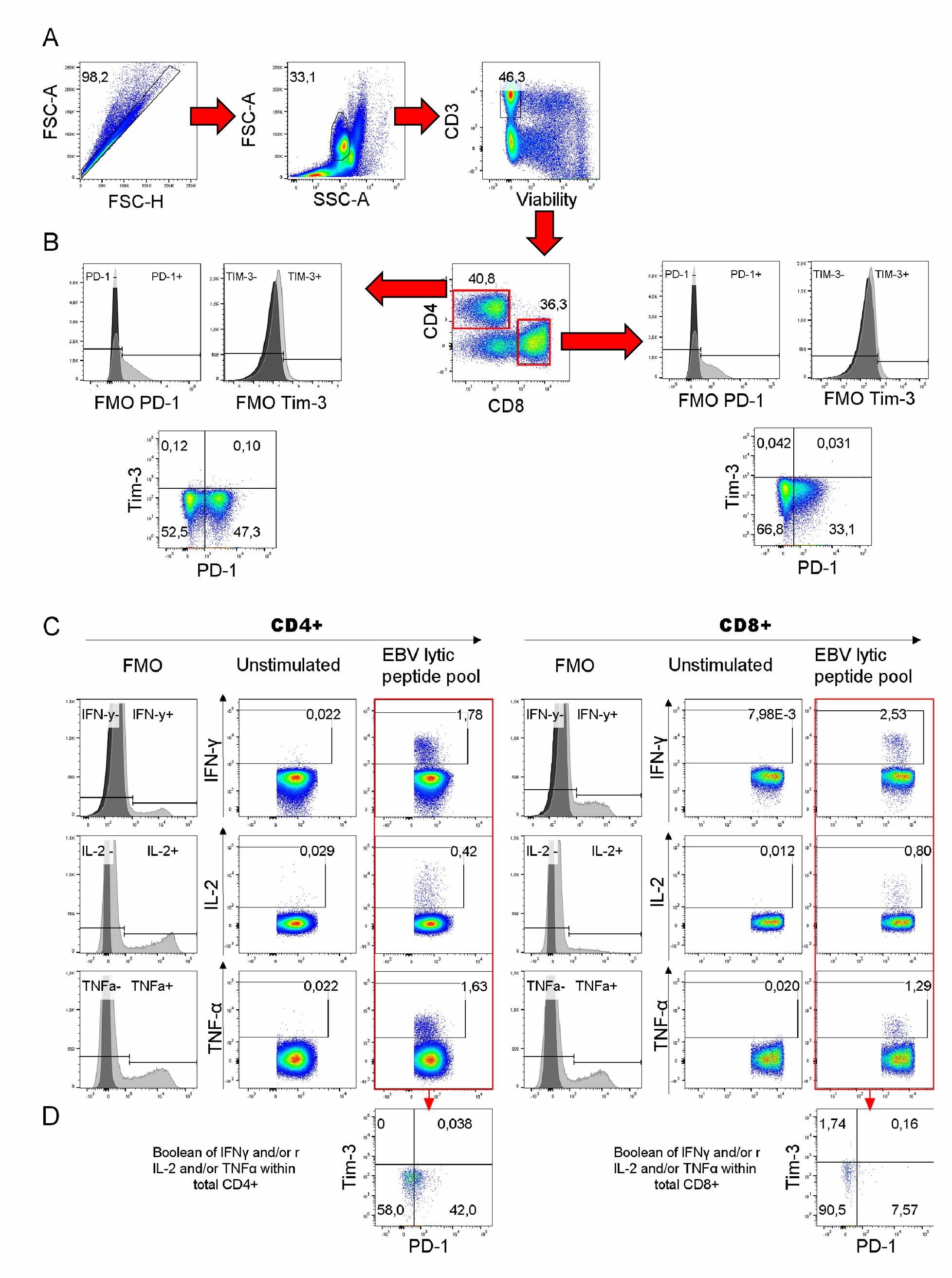

Supplement: S2 Fig — (A) Live CD3+ T cells were selected within total lymphocytes after doublet exclusion. (B) PD-1 and Tim-3 expression by total CD4+ and CD8+ T cells was measured under unstimulated condition according to FMO controls. (C) EBV-specific T cells were detected by cytokine production (IFNγ, IL-2, TNFα) out of total CD4+ and CD8+ T cells and pooled in a (D) single boolean gate to measure PD-1 and Tim-3 expression and co-expression. (TIF) [file pone.0224211.s002.tif]

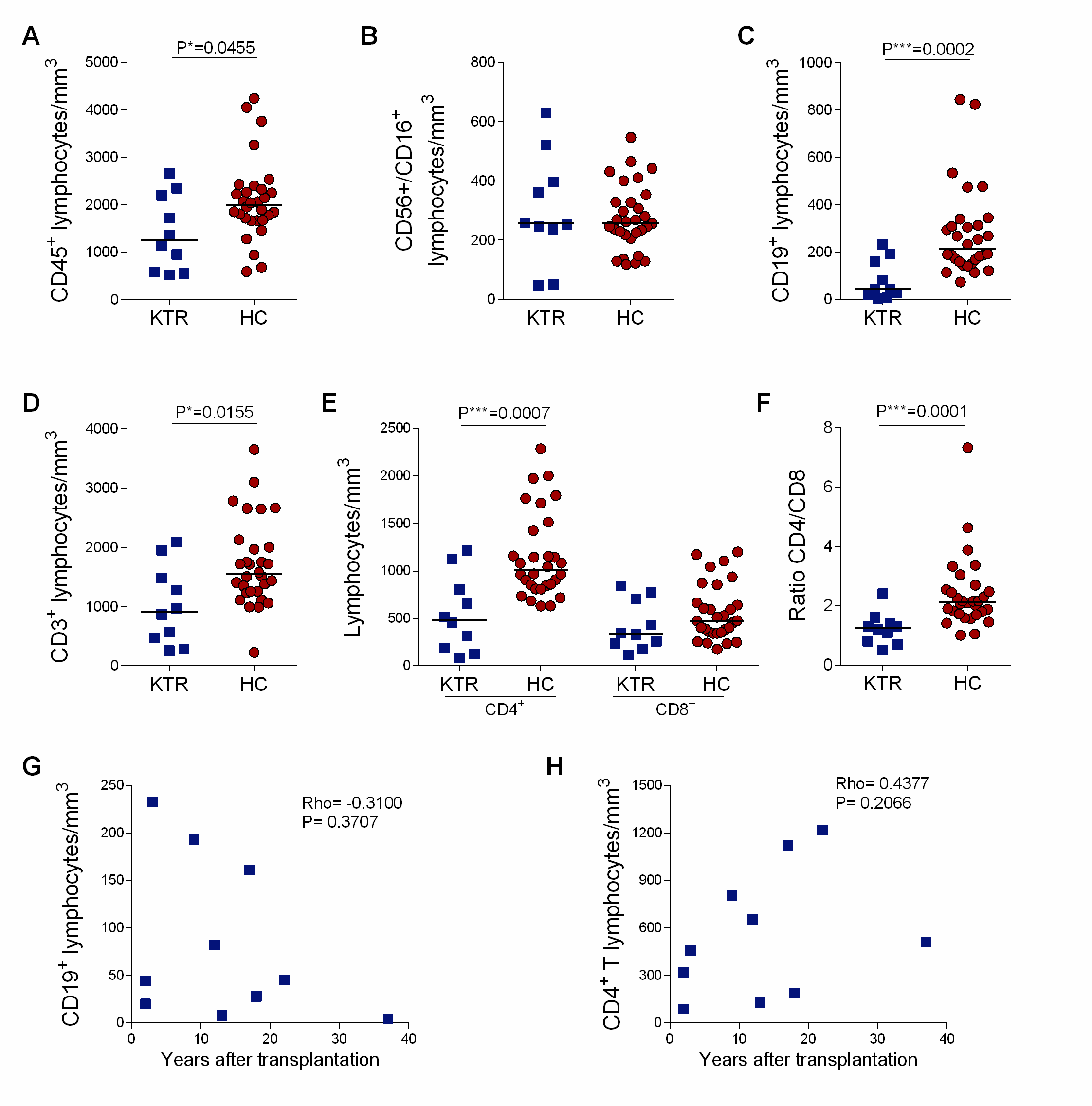

Supplement: S3 Fig — Absolute numbers of: (A) CD45+ lymphocytes, (B) CD3-CD56+/CD16+ NK cells, (C) CD19+ B cells; (D) CD3+ T cells; (E) CD4+ and CD8+ T cells; and (F) CD4/CD8 ratio from 10 kidney transplant recipients (KTRs) and 30 healthy controls(HCs). Horizontal bars indicate the median. Correlations between absolute counts of (G) CD19+ or (H) CD4+ T lymphocytes and the number of years after transplantation in 10 KTRs. Exact P-values were calculated with a two-tailed Mann-Whitney test and correlation was assessed with the Spearman rank correlation coefficient. Bonferroni significativity threshold for correlations was 0.0041. (TIF) [file pone.0224211.s003.tif]

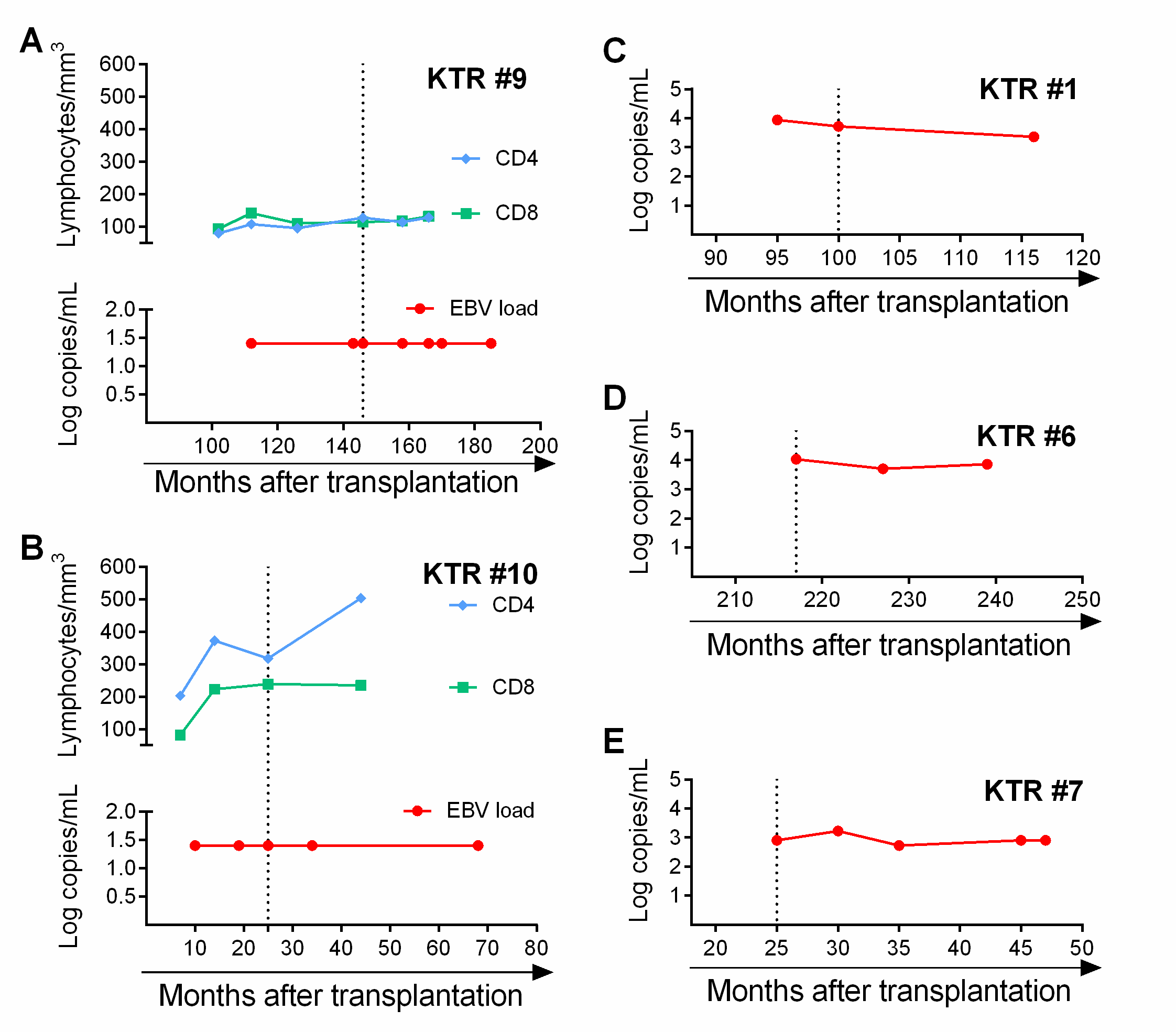

Supplement: S4 Fig — Absolute numbers of CD4+ and CD8+ T cells and EBV loads (Log copies/mL) at different time points in two kidney transplant recipients (KTRs): (A) KTR #9 and (B) KTR #10. EBV loads (Log copies/mL) at different time points in (C) KTR #1, (D) KTR #6 and (D) KTR #7. Dotted lines indicate the blood sample in this study. EBV load detection limit is 1.4 log copies/mL. (TIF) [file pone.0224211.s004.tif]

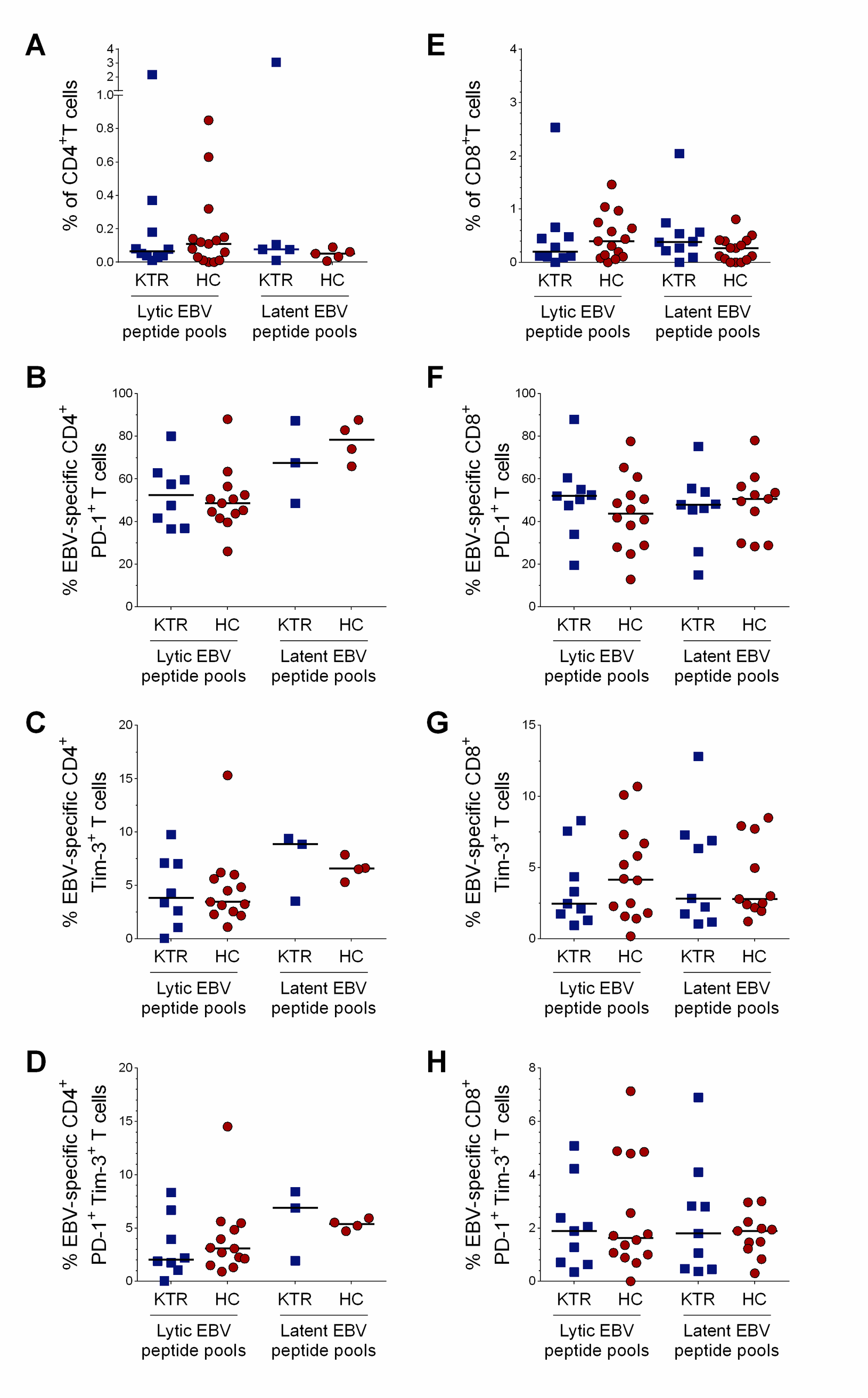

Supplement: S5 Fig — (A) Frequency of latent (KTRs n = 5; HCs n = 5) and lytic (KTRs n = 10; HCs n = 15) EBV-specific CD4+ T cells (IFNγ, IL-2, and TNFα) determined by intracellular cytokine staining assay with flow cytometry. Frequency of (B) PD-1 and (C) Tim-3 expression and (D) co-expression on latent (responders: KTRs n = 3/5; HCs n = 4/5) and lytic (responders: KTRs n = 8/10; HCs n = 13/15) EBV-specific CD4+ T cells from responding KTRs and HCs. (E) Frequency of latent and lytic EBV-specific CD8+ T cells (KTRs n = 10; HCs n = 15). Frequency of (F) PD-1 and (G) Tim-3 expression and (H) co-expression on latent (responders: KTRs n = 9/10; HCs n = 11/15) and lytic (responders: KTRs n = 9/10; HCs n = 14/15) EBV-specific CD8+ T cells from responding KTRs and HCs. Horizontal bars indicate the median. Exact P-values were calculated with a two-tailed Mann-Whitney test; only significant values (P<0.05) are shown. (TIF) [file pone.0224211.s005.tif]

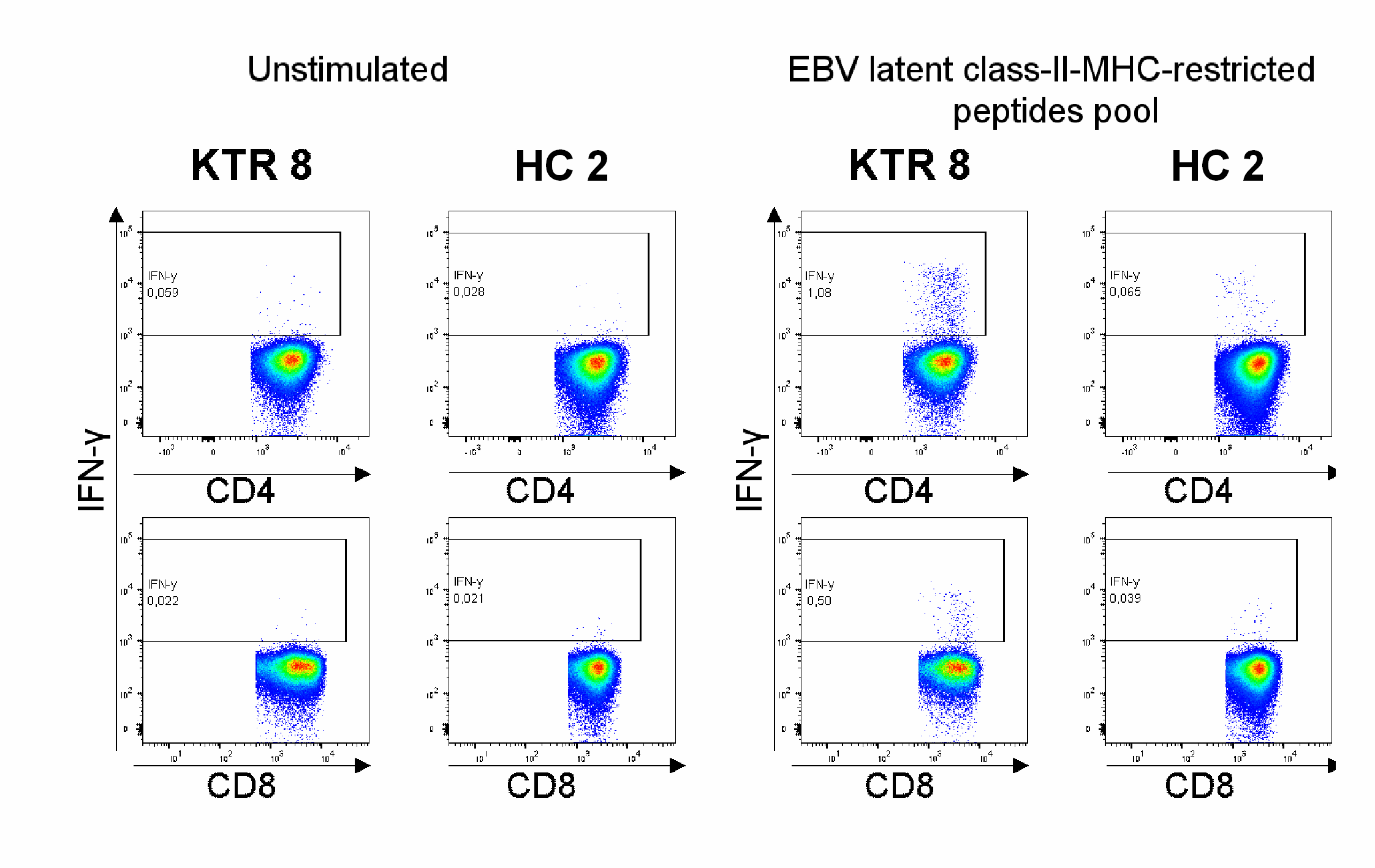

Supplement: S6 Fig — PBMCs of one kidney transplant recipient (KTR 8) and one healthy control (HC 2), were stimulated with media or with latent EBV class II MHC-restricted peptides to verify the specificity of peptide recognition. Dot plot shows IFNγ+ cells within total CD4+ or CD8+ T cells detected by intracellular cytokine (IFNγ) staining assay in flow cytometry. (TIF) [file pone.0224211.s006.tif]

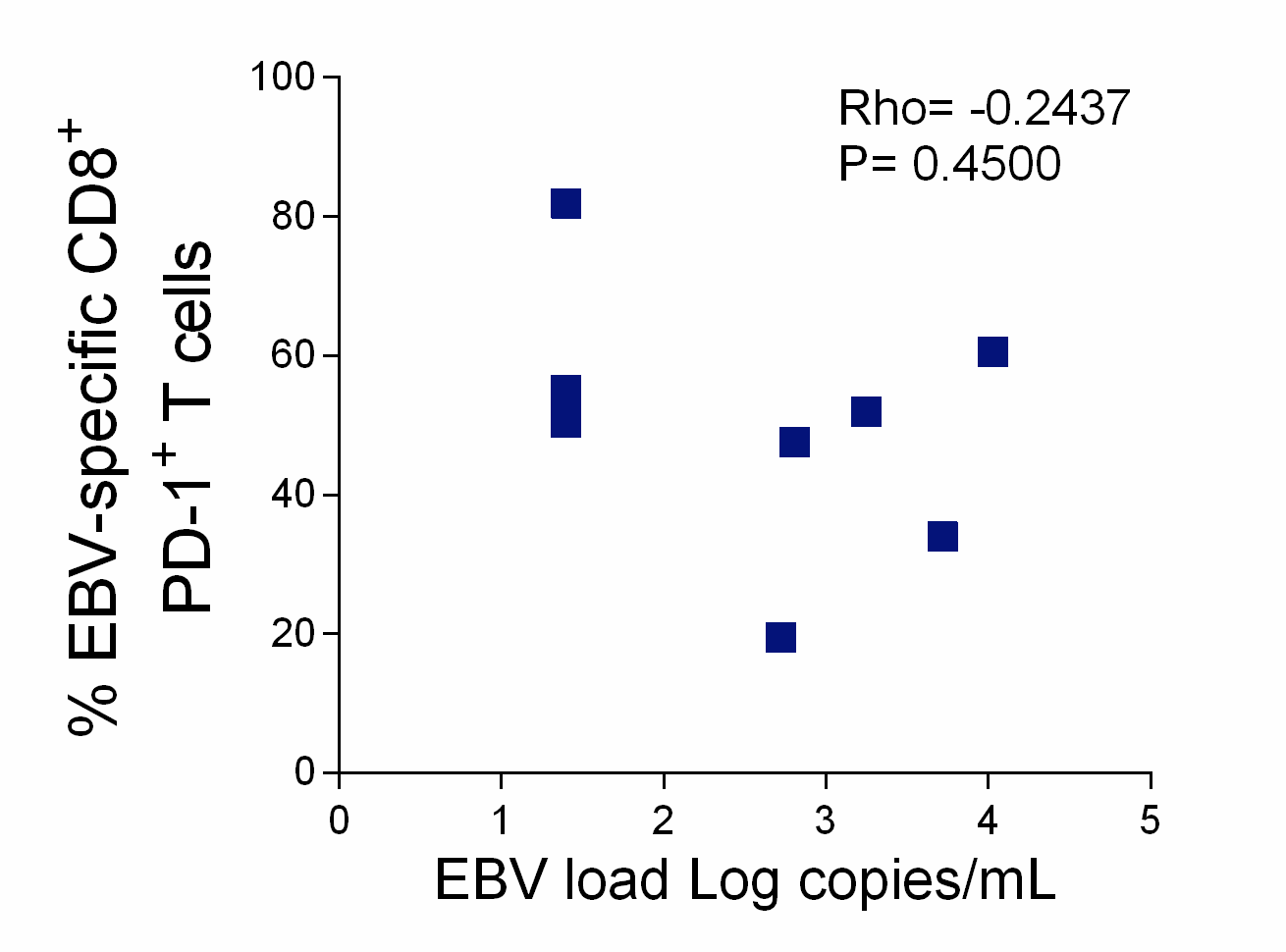

Supplement: S7 Fig — The correlation between the frequency of PD-1 expression on lytic-EBV-specific CD8+ T cells from responding KTRs (n = 9/10) and the EBV load (Log of copies/mL) was assessed with the Spearman rank correlation coefficient. Bonferroni significativity threshold was 0.0041. (TIF) [file pone.0224211.s007.tif]

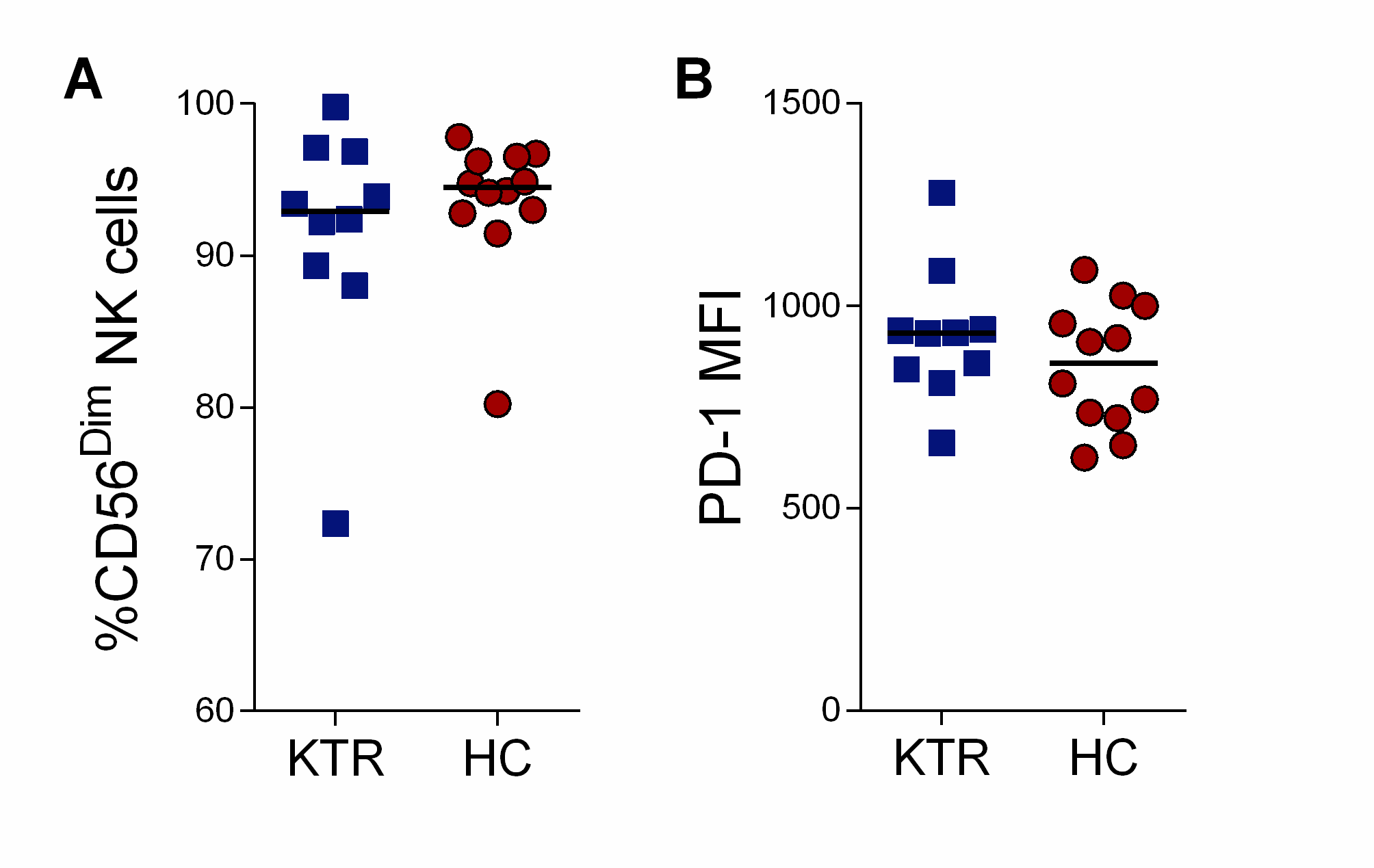

Supplement: S8 Fig — Percentage of (A) CD56Dim CD3- NK cells and (B) mean fluorescence intensity (MFI) of PD-1 in total CD56+CD3- NK cells from 10 kidney transplant recipients (KTRs) and 12 healthy controls (HCs). Expression was measured at the surface by flow cytometry on thawed PBMCs. Horizontal bars indicate the median. Exact P-values were calculated with a two-tailed Mann-Whitney test. (TIF) [file pone.0224211.s008.tif]

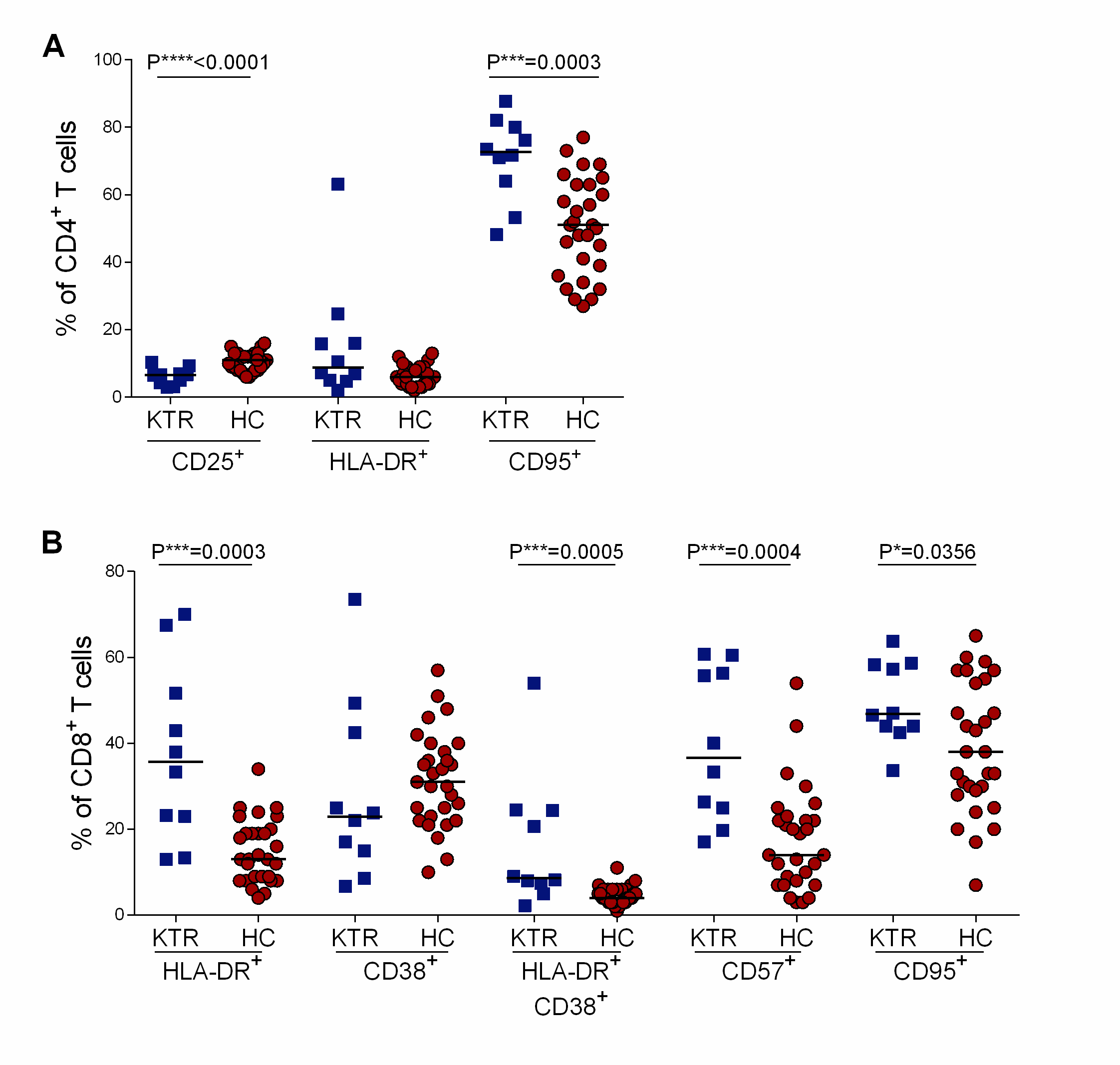

Supplement: S9 Fig — Frequency of (A) CD4+ and (B) CD8+ T cells expressing common activation markers detected by flow cytometry. Data are shown for kidney transplant recipients (KTRs; n = 10) and healthy controls (HCs; n = 30). Horizontal bars indicate the median. Exact P-values were calculated with a two-tailed Mann-Whitney test. (TIF) [file pone.0224211.s009.tif]

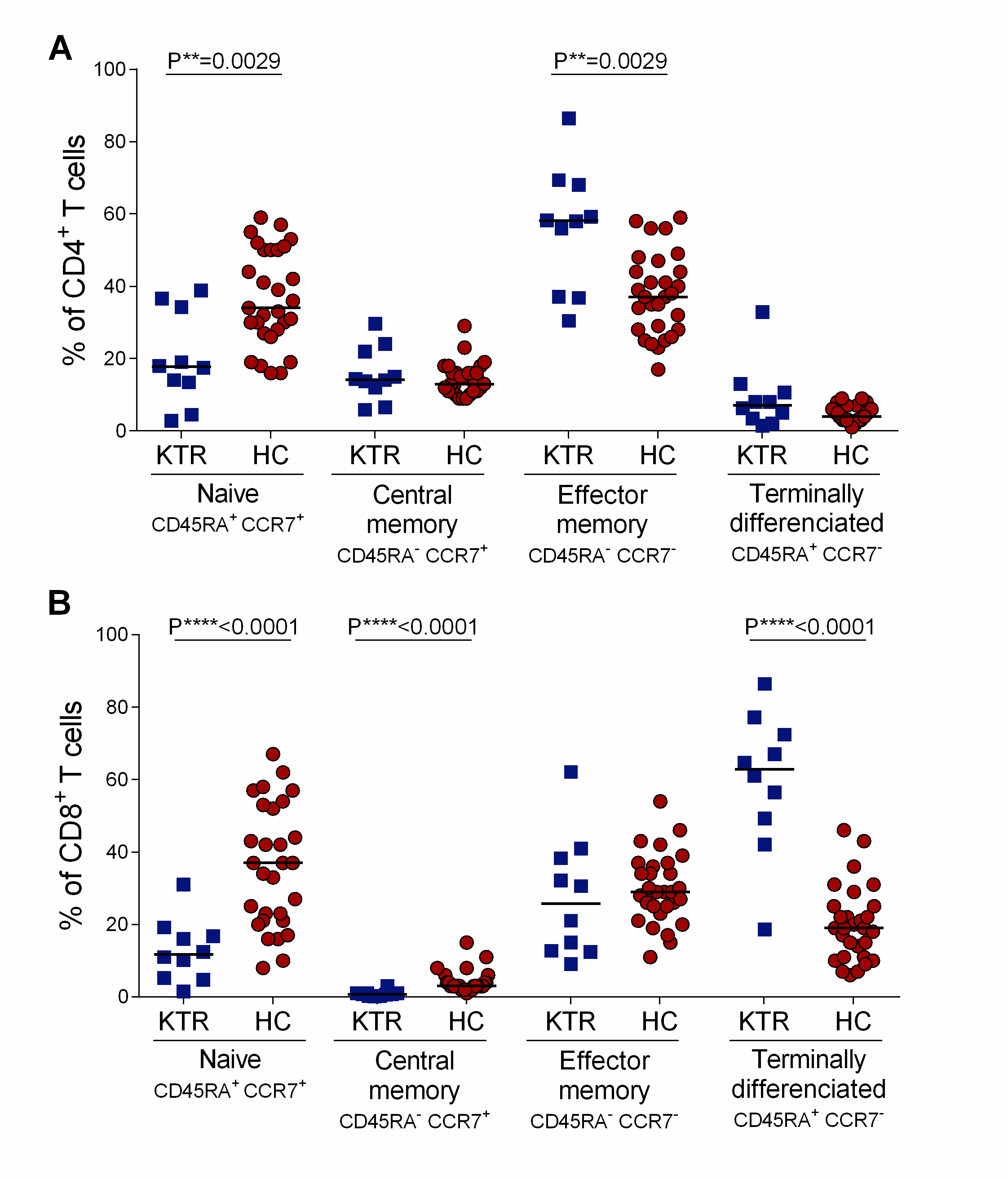

Supplement: S10 Fig — Frequency of (A) CD4+ and (B) CD8+ T cell differentiation subtypes detected by flow cytometry. Data are shown for kidney transplant recipients (KTRs; n = 10) and healthy controls (HCs; n = 30). Horizontal bars indicate the median. Exact P-values were calculated with a two-tailed Mann-Whitney test. (TIF) [file pone.0224211.s010.tif]
