# Supplementary material for: Skewed T cell responses to Epstein-Barr virus in long-term asymptomatic kidney transplant recipients
Source: PLoS One. 2019 Oct 22;14(10):e0224211. doi: 10.1371/journal.pone.0224211 (PMC6804993; doi:10.1371/journal.pone.0224211)
Supplement: S1 Table — (PDF) [file pone.0224211.s011.pdf]

S1 Table. Sequences of 42 class I MHC-restricted latent and lytic EBV optimal peptides.

Protein	Epitope	HLA	Sequence	Pool
EBNA-3A	246–253	A24	RYSIFFDY	1
EBNA-3A	603–611	A3	RLRAEAQVK	1
EBNA-3A	458–466	B35	YPLHEQHGM	1
EBNA-3A	406–414	B62	LEKARGSTY	1
EBNA-3A	379–387	B7	RPPIFIRRL	1
EBNA-3A	502–510	B7	VPAPAGPIV	1
EBNA-3A	325–333	B8	FLRGRAYGL	1
EBNA-3B	399–408	A11	AVFDRKSDAK	1
EBNA-3A	158–166	B8	QAKWRLQTL	2
EBNA-3B	416–424	A11	IVTDFSVIK	2
EBNA-3B	217–225	A24.02	TYSAGIVQI	2
EBNA-3B	657–666	B44	VEITPYKPTW	2
EBNA-3B	831–839	B62	GQGGSPTAM	2
EBNA-3C	284–293	A2.01	LLDFVRFMGV	2
EBNA-3C	881–889	B7	QPRAPIRPI	2
LMP-2	340-350	A11	SSCSSCPLSKI	2
LMP-2	329-337	A2.01	LLWTLVVLL	2
LMP-2	419-427	A24	TYGPVFMCL	2
EBNA-3A	596-604	A2	SVRDRLARL	3
EBNA-3A	491-499	A29	VFSDGRVAC	3
EBNA-3A	176-184	A30.02	AYSSWMYSY	3
EBNA-3B	244-254	B27.02	RRARSLSAERY	3
EBNA-3B	149-157	B27.05	HRCQAIRKK	3
EBNA-3B	488-496	B35	AVLLHEESM	3
EBNA-3C	258-266	B27.02/.04/.05	RRIYDLIEL	3
EBNA-3C	249-258	B27.05	LRGKWQRRYR	3
EBNA-3C	271-278	B39	HHIWQNLL	3
EBNA-1	407-417	B35.01	HPVGEADYFEY	3
EBNA-3C	163-171	B44.03	EGGVGWRHW	3
EBNA-3C	343-351	B27.05	FRKAQIQGL	4
EBNA-3C	281-290	B44.02	EENLLDFVRF	4
EBNA-3C	335-343	B44.02	KEHVIQNAF	4
EBNA-3C	213-222	B62	QNGALAINTF	4
LMP-2	426-434	A2.01	CLGGLLTMV	4
LMP-2	453-461	A2.06	LTAGFLIFL	4
LMP-2	131-139	A23	PYLFWLAAI	4
LMP-2	442-451	A25	VMSNTLLSAW	4
LMP-2	236-244	B27.04	RRRWRRLTV	4
LMP-2	200-208	B40	IEDPPFNSL	4
BMLF-1	280-288	A2.01	GLCTLVAML	5
BZLF-1	54-64	B35	EPLPQGQLTAY	5
BZLF-1	190-197	B8	RAKFKQLL	5
