# Supplementary material for: Skewed T cell responses to Epstein-Barr virus in long-term asymptomatic kidney transplant recipients
Source: PLoS One. 2019 Oct 22;14(10):e0224211. doi: 10.1371/journal.pone.0224211 (PMC6804993; doi:10.1371/journal.pone.0224211)
Supplement: S2 Table — (PDF) [file pone.0224211.s012.pdf]

S2 Table. Sequences of 33 class II MHC-restricted latent EBV optimal peptides.

Protein	Epitope	HLA	Peptide sequence	Pool
BHRF1	171-189	DR2	AGLTLSLLVICSYLFISRG	1
EBNA-1	475-489	DR11	NPKFENIAEGLRALL	1
EBNA-1	71-85	DR11, DR13, DQ3, DQ6	RRPQKRPSCIGCKGT	1
EBNA-1	485-499	DR11, DR13, DR15, DQ1, DQ3, DQ6	LRALLARSHVERTTD	1
EBNA-1	515-527	DR1	TSLYNLRRGTALA	1
EBNA-1	529-543	DR13, DQ3, DQ5, DQ6	PQCRLTPLSRLPFGM	1
EBNA-1	429-448	DR1, DR3, DQ2, DQ5	VPPGAIEQGPADDPGEGPST	1
EBNA-1	455-469	DR 7, DR15, DQ1, DQ2	DGGRRKKGGWFGRHR	1
EBNA-1	509-528	DR11, DR13, DQ3, DQ6	VYGGSKTSLYNLRRGTALAI	1
EBNA-1	544-563	DR1, DR3, DQ2, DQ5	APGPGPQPGPLRESIVCYFM	1
EBNA-1	554-573	DR1, DR3, DQ2, DQ5	LRESIVCYFMVFLQTHIFAE	1
EBNA-1	574-593	DR1, DR3, DQ2, DQ5	VLKDAIKDLVMTKPAPTCNI	2
EBNA-1	594-613	ND	RVTVCSFDDGVDLPPWFPPM	2
EBNA-1	519-533	DR4, DQ3	NLRRGTALAIPQCRL	2
EBNA-1	563-577	DR15	MVFLQTHIFAEVLKD	2
EBNA-1	514-533	DR1	KTSLYNLRRGIALAIPQCRL	2
EBNA1	424-443	DR1 , DR4, DQ1, DQ8	DGEPDMPPGAIEQGPADDPG	2
EBNA-1	403-417	DQ2	RPFFHPVGEADYFEY	2
EBNA-1	589-608	DR1	PTCNIKATVCSFDDGVDLPP	2
EBNA-2	280-290	DQ2	TVFYNIPPMPL	2
EBNA-3C	386-400	DR1, DR13, DQ5, DQ6	SDDELPYIDPNMEPV	2
EBNA-3C	961-986	DR4, DQ2, DQ3, DQ5	AQEILSDNSEISVFPK	2
EBNA-3C	141-155	DR1, DR13, DQ5, DQ6	ILCFVMAARQRLQDI	3
EBNA-3C	401-415	DR11, DR14, DQ3, DQ5	QQRPVMFVSRVPAKK	3
EBNA-3C	546-560	DR1, DR13, DQ5, DQ6	QKRAAPPTVSPSDTG	3
EBNA-3C	916-930	DR4	PSMPFASDYSQGAFT	3
EBNA-3C	626-640	DR11, DR14, DQ3, DQ5	PPVVRMFMRERQLPQ	3
EBNA-3C	646-660	ND	PQCFWEMRAGREITQ	3
EBNA-3C	586-600	DR1, DR13, DQ5, DQ6	PPAAGPPAAGPRILA	3
LMP-1	130-144	DR1, DR4, DQ1, DQ3	LWRLGATIWQLLAFF	3
LMP-1	340-354	ND	TDGGGGHSHDSGHGG	3
LMP-1	212-226	DQ2	SGHESDSNSNEGRHH	3
LMP-2A	385-398	ND	STEFIPNLFCMLLL	3

ND, not determined.
