# Supplementary material for: Skewed T cell responses to Epstein-Barr virus in long-term asymptomatic kidney transplant recipients
Source: PLoS One. 2019 Oct 22;14(10):e0224211. doi: 10.1371/journal.pone.0224211 (PMC6804993; doi:10.1371/journal.pone.0224211)
Supplement: S3 Table — 47 15mer peptides overlapping by 10 amino acids and covering the lytic BZLF-1 protein. (PDF) [file pone.0224211.s013.pdf]

S3 Table. Sequences of BZLF-1 overlapping peptides

47 15mer peptides overlapping by 10 amino acids and covering the lytic BZLF-1 protein

Protein	Location	Peptide sequence	Pool
BZLF-1	1-15	MMDPNSTSEDVKFTP	1
BZLF-1	6-20	STSEDVKFTPDPYQV	1
BZLF-1	11-25	VKFTPDPYQVPFVQA	1
BZLF-1	16-30	DPYQVPFVQAFDQAT	1
BZLF-1	21-35	PFVQAFDQATRVYQD	1
BZLF-1	26-40	FDQATRVYQDLGGPS	1
BZLF-1	31-45	RVYQDLGGPSQAPLP	1
BZLF-1	36-50	LGGPSQAPLPCVLWP	1
BZLF-1	41-55	QAPLPCVLWPVLPEP	1
BZLF-1	46-60	CVLWPVLPEPLPQGQ	1
BZLF-1	51-65	VLPEPQGQLTAYH	2
BZLF-1	56-70	LPQGQLTAYHVSTAP	2
BZLF-1	61-75	LTAYHVSTAPTGSWF	2
BZLF-1	66-80	VSTAPTGWFSAPQP	2
BZLF-1	71-85	TGSWFSAPQPAPENA	2
BZLF-1	76-90	SAPQPAPENAYQAYA	2
BZLF-1	81-95	APENAYQAYAAPQLF	2
BZLF-1	86-100	YQAYAAPQLFPVSDI	2
BZLF-1	91-105	APQLFPVSDITQNQQ	2
BZLF-1	96-110	PVSDITQNQQTNQAG	2
BZLF-1	101-115	TQNQQTNQAGGEAPQ	3
BZLF-1	106-120	TNQAGGEAPQPGDNS	3
BZLF-1	111-125	GEAPQPGDNSTVQTA	3
BZLF-1	116-130	PGDNSTVQTAAAVVF	3
BZLF-1	121-135	TVQTAAAVVFACPGA	3
BZLF-1	126-140	AAVVFACPGANQGQQ	3
BZLF-1	131-145	ACPGANQGQQLADIG	3
BZLF-1	136-150	NQGQQLADIGVPQPA	3
BZLF-1	141-155	LADIGVPQPAPVAAP	3
BZLF-1	146-160	VPQAPVAAPARRTR	3
BZLF-1	151-165	PVAAPARRTRKPQQP	4
BZLF-1	156-170	ARRTRKPQQPESLEE	4
BZLF-1	161-175	KPQQPESLEECDSEL	4
BZLF-1	166-180	ESLEECDSELEIKRY	4
BZLF-1	171-185	CDSELEIKRYKNRVA	4
BZLF-1	176-190	EIKRYKNRVASRKCR	4
BZLF-1	181-195	KNRVASRKCRAKFKQ	4
BZLF-1	186-200	SRKCRAKFKQLLQHY	4
BZLF-1	191-205	AKFKQLLQHYREVAA	4
BZLF-1	196-210	LLQHYREVAAAKSSE	4
BZLF-1	201-215	REVAAAKSSENDRLR	5
BZLF-1	206-220	AKSSENDRLRLLLKQ	5
BZLF-1	211-225	NDRLRLLLKQMCPSL	5
BZLF-1	216-230	LLLKQMCPSLDVDSI	5
BZLF-1	221-235	MCPSLDVDSIIPRTP	5

BZLF-1	226-240	DVDSIIPRTPDVLHE	5
BZLF-1	230-245	IPRTPDVLHEDLLNF	5
