# Supplementary material for: Skewed T cell responses to Epstein-Barr virus in long-term asymptomatic kidney transplant recipients
Source: PLoS One. 2019 Oct 22;14(10):e0224211. doi: 10.1371/journal.pone.0224211 (PMC6804993; doi:10.1371/journal.pone.0224211)
Supplement: S4 Table — 160 15mer peptides overlapping by 10 amino acids and partially covering the latent EBNA-3A protein. (PDF) [file pone.0224211.s014.pdf]

**S4 Table. Sequences of EBNA-3A overlapping peptides**			
160 15mer peptides overlapping by 10 amino acids and partially covering the latent EBNA-3A protein			
**Protein**	**Location**	**Peptide sequence**	**Pool**
EBNA-3A	133 - 147	MYIMY AMAIR QAIRD	1
EBNA-3A	138 - 152	AMAIR QAIRD RRRNP	1
EBNA-3A	143 - 157	QAIRD RRRNP ASRRD	1
EBNA-3A	148 - 162	RRRNP ASRRD QAKWR	1
EBNA-3A	153 - 167	ASRRD QAKWR LQTLA	1
EBNA-3A	158 - 172	QAKWR LQTLA AGWPM	1
EBNA-3A	163 - 177	LQTLA AGWPM GYQAY	1
EBNA-3A	168 - 182	AGWPM GYQAY SSWMY	1
EBNA-3A	173 - 187	GYQAY SSWMY SYTDH	1
EBNA-3A	178 - 192	SSWMY SYTDH QTTPT	1
EBNA-3A	183 - 197	SYTDH QTTPT FVHLQ	2
EBNA-3A	188 - 202	QTTPT FVHLQ ATLGC	2
EBNA-3A	193 - 207	FVHLQ ATLGC TGGRR	2
EBNA-3A	198 - 212	ATLGC TGGRR CHVTF	2
EBNA-3A	203 - 217	TGGRR CHVTF SAGTF	2
EBNA-3A	208 - 222	CHVTF SAGTF KLPRC	2
EBNA-3A	213 - 227	SAGTF KLPRC TPGDR	2
EBNA-3A	218 - 232	KLPRC TPGDR QWLYV	2
EBNA-3A	223 - 237	TPGDR QWLYV QSSVG	2
EBNA-3A	228 - 242	QWLYV QSSVG NIVQS	2
EBNA-3A	233 - 247	QSSVG NIVQS CNPRY	3
EBNA-3A	238 - 252	NIVQS CNPRY SIFFD	3
EBNA-3A	243 - 257	CNPRY SIFFD YMAIH	3
EBNA-3A	248 - 262	SIFFD YMAIH RSLTK	3
EBNA-3A	253 - 267	YMAIH RSLTK IWEEV	3
EBNA-3A	258 - 272	RSLTK IWEEV LTPDQ	3
EBNA-3A	263 - 277	IWEEV LTPDQ RVSFM	3
EBNA-3A	268 - 282	LTPDQ RVSFM EFLGF	3
EBNA-3A	273 - 287	RVSFM EFLGF LQRTD	3
EBNA-3A	278 - 292	EFLGF LQRTD LSYIK	3
EBNA-3A	283 - 297	LQRTD LSYIK SFVSD	4
EBNA-3A	288 - 302	LSYIK SFVSD ALGTT	4
EBNA-3A	293 - 307	SFVSD ALGTT SIQTP	4
EBNA-3A	298 - 312	ALGTT SIQTP WIDDN	4
EBNA-3A	303 - 317	SIQTP WIDDN PSTET	4
EBNA-3A	308 - 322	WIDDN PSTET AQAWN	4
EBNA-3A	313 - 327	PSTET AQAWN AGFLR	4
EBNA-3A	318 - 332	AQAWN AGFLR GRAYG	4
EBNA-3A	323 - 337	AGFLR GRAYG IDLLR	4

EBNA-3A	328 - 342	GRAYG IDLLR TEGEH	4
EBNA-3A	333 - 347	IDLLR TEGEH VEGAT	5
EBNA-3A	338 - 352	TEGEH VEGAT GETRE	5
EBNA-3A	343 - 357	VEGAT GETRE ESEDT	5
EBNA-3A	348 - 362	GETRE ESEDT ESDGD	5
EBNA-3A	353 - 367	ESEDT ESDGD DEDELP	5
EBNA-3A	358 - 372	ESDGD DEDELP CIVSR	5
EBNA-3A	363 - 377	DEDELP CIVSR GGPKV	5
EBNA-3A	368 - 382	CIVSR GGPKV KRPPI	5
EBNA-3A	373 - 387	GGPKV KRPPI FIRRL	5
EBNA-3A	378 - 392	KRPPI FIRRL HRLLL	5
EBNA-3A	383 - 397	FIRRL HRLLL MRAGK	6
EBNA-3A	388 - 402	HRLLL MRAGK RTEQG	6
EBNA-3A	393 - 407	MRAGK RTEQG KEVLE	6
EBNA-3A	398 - 412	RTEQG KEVLE KARGS	6
EBNA-3A	403 - 417	KEVLE KARGS TYGTP	6
EBNA-3A	408 - 422	KARGS TYGTP RPPVP	6
EBNA-3A	413 - 427	TYGTP RPPVP KPRPE	6
EBNA-3A	418 - 432	RPPVPKPRPEVPQSD	6
EBNA-3A	423 - 437	KPRPE VPQSD ETATS	6
EBNA-3A	428 - 442	VPQSD ETATS HGSAQ	6
EBNA-3A	433 - 447	ETATS HGSAQ VPEPP	7
EBNA-3A	438 - 452	HGSAQ VPEPP TIHLA	7
EBNA-3A	443 - 457	VPEPP TIHLA AQGMA	7
EBNA-3A	448 - 462	TIHLA AQGMA YPLHE	7
EBNA-3A	453 - 467	AQGMA YPLHE QHGMA	7
EBNA-3A	458 - 472	YPLHE QHGMA PCPVA	7
EBNA-3A	463 - 477	QHGMA PCPVA QAPPT	7
EBNA-3A	468 - 482	PCPVA QAPPT PLPPV	7
EBNA-3A	473 - 487	QAPPT PLPPV SPGDQ	7
EBNA-3A	478 - 492	PLPPV SPGDQ LPGVF	7
EBNA-3A	483 - 497	SPGDQ LPGVF SDGRV	8
EBNA-3A	488 - 502	LPGVF SDGRV ACAPV	8
EBNA-3A	493 - 507	SDGRV ACAPV PAPAG	8
EBNA-3A	498 - 512	ACAPV PAPAG PIVRP	8
EBNA-3A	503 - 517	PAPAG PIVRP WEPSL	8
EBNA-3A	508 - 522	PIVRP WEPSL TQAAG	8
EBNA-3A	513 - 527	WEPSL TQAAG QAFAP	8
EBNA-3A	518 - 532	TQAAG QAFAP VRPQH	8
EBNA-3A	523 - 537	QAFAP VRPQH MPVEP	8
EBNA-3A	528 - 542	VRPQH MPVEP VPVPT	8
EBNA-3A	533 - 547	MPVEP VPVPT VALER	9
EBNA-3A	538 - 552	VPVPT VALER PVYPK	9
EBNA-3A	543 - 557	VALER PVYPK PVRPA	9

EBNA-3A	548	-	562	PVYPK PVRPA PPLIA	9
EBNA-3A	553	-	567	PVRPA PPLIA MQGPG	9
EBNA-3A	558	-	572	PPLIA MQGPG ETSGI	9
EBNA-3A	563	-	577	MQGPG ETSGI RRARE	9
EBNA-3A	568	-	582	ETSGI RRARE RWRPA	9
EBNA-3A	573	-	587	RRARE RWRPA PWTPN	9
EBNA-3A	578	-	592	RWRPA PWTPN PPRSP	9
EBNA-3A	583	-	597	PWTPN PPRSP SQMSV	10
EBNA-3A	588	-	602	PPRSP SQMSV RDRLA	10
EBNA-3A	593	-	607	SQMSV RDRLA RLRAE	10
EBNA-3A	598	-	612	RDRLA RLRAE AQVKQ	10
EBNA-3A	603	-	617	RLRAE AQVKQ ASVEV	10
EBNA-3A	608	-	622	AQVKQ ASVEV QPPQL	10
EBNA-3A	613	-	627	ASVEV QPPQL TQVSP	10
EBNA-3A	618	-	632	QPPQL TQVSP QQPME	10
EBNA-3A	623	-	637	TQVSP QQPME GPLVP	10
EBNA-3A	628	-	642	QQPME GPLVP EQQMF	10
EBNA-3A	633	-	647	GPLVP EQQMF PGAPF	11
EBNA-3A	638	-	652	EQQMF PGAPF SQVAD	11
EBNA-3A	643	-	657	PGAPF SQVAD VVRAP	11
EBNA-3A	648	-	662	SQVAD VVRAP GVPAM	11
EBNA-3A	653	-	667	VVRAP GVPAM QPQYF	11
EBNA-3A	658	-	672	GVPAM QPQYF DLPLI	11
EBNA-3A	663	-	677	QPQYF DLPLI QPISQ	11
EBNA-3A	668	-	682	DLPLI QPISQ GAPVA	11
EBNA-3A	673	-	687	QPISQ GAPVA PLRAS	11
EBNA-3A	678	-	692	GAPVA PLRAS MGPVP	11
EBNA-3A	683	-	697	PLRAS MGPVP PVPAT	12
EBNA-3A	688	-	702	MGPVP PVPAT QPQYF	12
EBNA-3A	693	-	707	PVPAT QPQYF DIPLT	12
EBNA-3A	698	-	712	QPQYF DIPLT EPINQ	12
EBNA-3A	703	-	717	DIPLT EPINQ GASAA	12
EBNA-3A	708	-	722	EPINQ GASAA HFLPQ	12
EBNA-3A	713	-	727	GASAA HFLPQ QPMEG	12
EBNA-3A	718	-	732	HFLPQ QPMEG PLVPE	12
EBNA-3A	723	-	737	QPMEG PLVPE QWMFP	12
EBNA-3A	728	-	742	PLVPE QWMFP GAALS	12
EBNA-3A	733	-	747	QWMFP GAALS QSVRP	13
EBNA-3A	738	-	752	GAALS QSVRP GVAQS	13
EBNA-3A	743	-	757	QSVRP GVAQS QYFDL	13
EBNA-3A	748	-	762	GVAQS QYFDL PLTQP	13
EBNA-3A	753	-	767	QYFDL PLTQP INHGA	13
EBNA-3A	758	-	772	PLTQP INHGA PAAHF	13
EBNA-3A	763	-	777	INHGA PAAHF LHQPP	13

EBNA-3A	768 - 782	PAAHF LHQPP MEGPW	13
EBNA-3A	773 - 787	LHQPP MEGPW VPEQW	13
EBNA-3A	778 - 792	MEGPW VPEQW MFQGA	13
EBNA-3A	783 - 797	VPEQW MFQGA PPSQG	14
EBNA-3A	788 - 802	MFQGA PPSQG TDVVQ	14
EBNA-3A	793 - 807	PPSQG TDVVQ HQLDA	14
EBNA-3A	798 - 812	TDVVQ HQLDA LGYTL	14
EBNA-3A	803 - 817	HQLDA LGYTL HGLNH	14
EBNA-3A	808 - 822	LGYTL HGLNH PGVPV	14
EBNA-3A	813 - 827	HGLNH PGVPV SPAVN	14
EBNA-3A	818 - 832	PGVPV SPAVN QYHLS	14
EBNA-3A	823 - 837	SPAVN QYHLS QAAFG	14
EBNA-3A	828 - 842	QYHLS QAAFG LPIDE	14
EBNA-3A	833 - 847	QAAFG LPIDE DESGE	15
EBNA-3A	838 - 852	LPIDE DESGE GSDTS	15
EBNA-3A	843 - 857	DESGE GSDTS EPCEA	15
EBNA-3A	848 - 862	GSDTS EPCEA LDLSI	15
EBNA-3A	853 - 867	EPCEA LDLSI HGRPC	15
EBNA-3A	858 - 872	LDLSI HGRPC PQAPE	15
EBNA-3A	863 - 877	HGRPC PQAPE WPVQE	15
EBNA-3A	868 - 882	PQAPE WPVQE EGGQD	15
EBNA-3A	873 - 887	WPVQE EGGQD ATEVL	15
EBNA-3A	878 - 892	EGGQD ATEVL DLSIH	15
EBNA-3A	883 - 897	ATEVL DLSIH GRPRP	16
EBNA-3A	888 - 902	DLSIH GRPRP RTPEW	16
EBNA-3A	893 - 907	GRPRP RTPEW PVQGE	16
EBNA-3A	898 - 912	RTPEW PVQGE GGQNV	16
EBNA-3A	903 - 917	PVQGE GGQNV TGPET	16
EBNA-3A	908 - 922	GGQNV TGPET RRVVV	16
EBNA-3A	913 - 927	TGPET RRVVV SAVVH	16
EBNA-3A	918 - 932	RRVVV SAVVH MCQDD	16
EBNA-3A	923 - 937	SAVVH MCQDD EFPDL	16
EBNA-3A	928 - 944	MCQDD EFPDL QDPPDEA	16
