# Supplementary material for: Skewed T cell responses to Epstein-Barr virus in long-term asymptomatic kidney transplant recipients
Source: PLoS One. 2019 Oct 22;14(10):e0224211. doi: 10.1371/journal.pone.0224211 (PMC6804993; doi:10.1371/journal.pone.0224211)
Supplement: S5 Table — ■ Predicted and positive response; 〼Predicted and negative response; ●Unpredicted and positive response; □Predicted but undetermined response. (PDF) [file pone.0224211.s015.pdf]

S5 Table. Class I- and class II-HLA alleles and predicted responses to EBV optimal peptides of kidney transplant recipients (KTRs)

Patient ID	class I-HLA						class II-HLA			
	Patient alleles	pools					Patient alleles	pools		
		1	2	3	4	5		1	2	3
**KTR 1**	A 3-11 B 40-14	⧄	⧄				DR 15-4	□	□	□
**KTR 2**	A 28-32 B 07-40	⧄	⧄		●		n/a			
**KTR 3**	A 28-? B 57-70						DR 10-15	⧄	⧄	
**KTR 4**	A 02-11 B 27-44	⧄	⧄	■	■	■	DR 07-13	⧄	■	⧄
**KTR 5**	A 1-2 B 7-?	■	⧄	⧄	⧄	⧄	DR 17-7 DQ 2-0	⧄	⧄	⧄
**KTR 6**	A 23-30 B 07-49	□	□	□	□		DR 03-07	□	□	
**KTR 7**	A 02-68 B 70-51		⧄	⧄	⧄	⧄	DR 17-13 DQ 02-06	⧄	⧄	⧄
**KTR 8**	A 02-29 B 44-57		□	□	□	□	DR ?-07	■	●	●
**KTR 9**	A 1-2 B 35-51	□	□	□	□	□	DR 3-13	□	□	□
**KTR 10**	A 1-2 B 35-57		⧄	⧄	■	■	DR 1-0 DQ 5-?	⧄	⧄	⧄

■Predicted and positive response; ⧄Predicted and negative response;
●Unpredicted and positive response; □Predicted but undetermined response.
HLA, Human Leukocyte Antigen; n/a, not available.
