# Supplementary material for: Skewed T cell responses to Epstein-Barr virus in long-term asymptomatic kidney transplant recipients
Source: PLoS One. 2019 Oct 22;14(10):e0224211. doi: 10.1371/journal.pone.0224211 (PMC6804993; doi:10.1371/journal.pone.0224211)
Supplement: S6 Table — EBV, Epstein-Barr virus. aBonferroni significativity threshold ≤ 0.0041 (PDF) [file pone.0224211.s016.pdf]

S6 Table. Correlations between T cell phenotype, time after transplantation and EBV load of kidney transplant recipients

T cell subpopulations	Time post-transplant (years)		EBV load in Log copies/mL	
	**Rho**	**P-value[a]**	**Rho**	**P-value[a]**
% $CD95^+$ $CD4^+$ T cells	-0.4620	0.1735	0.1438	0.6900
% Naive $CD4^+$ T cells ($CD45RA^+CCR7^+$)	0.3161	0.3707	0.0187	0.9651
% Effector memory $CD4^+$ T cells ($CD45RA^-CCR7^-$)	0.1337	0.7127	0.0750	0.8386
% HLA-DR^+ $CD8^+$ T cells	0.3830	0.2726	0.4627	0.1779
% HLA-DR^+CD38^+ $CD8^+$ T cells	-0.2188	0.5284	0.6003	0.0721
% Naive $CD8^+$ T cells ($CD45RA^+CCR7^+$)	0.1398	0.6990	-0.2126	0.4936
% central memory $CD8^+$ T cells ($CD45RA^-CCR7^+$)	-0.1022	0.7166	0.0509	0.8880
% Terminally differentiated effector memory $CD8^+$ T cells ($CD45RA^+CCR7^-$)	0.2979	0.3992	0.3939	0.2580

EBV, Epstein-Barr virus. [a]Bonferroni significativity threshold ≤ 0.0041
